# Particle fusion of super-resolution data reveals the unit structure of Nup96 in Nuclear Pore Complex

**DOI:** 10.1038/s41598-023-39829-5

**Published:** 2023-08-16

**Authors:** Wenxiu Wang, Arjen Jakobi, Yu-Le Wu, Jonas Ries, Sjoerd Stallinga, Bernd Rieger

**Affiliations:** 1 Faculty of Applied Sciences, Delft University of Technology, Delft, The Netherlands; 2 Cell Biology and Biophysics Unit, European Molecular Biology Laboratory (EMBL), Heidelberg, Germany; 3 Department of Chromosome Biology, University of Vienna, Max-Perutz Labs, Center for Molecular Biology, Vienna, Austria

**Keywords:** Super-resolution microscopy, Imaging, Structure determination, Cell biology, Molecular biology

## Abstract

Single molecule localization microscopy offers resolution nearly down to the molecular level with specific molecular labelling, and is thereby a promising tool for structural biology. In practice, however, the actual value to this field is limited primarily by incomplete fluorescent labelling of the structure. This missing information can be completed by merging information from many structurally identical particles in a particle fusion approach similar to cryo-EM single-particle analysis. In this paper, we present a data analysis of particle fusion results of fluorescently labelled Nup96 nucleoporins in the Nuclear Pore Complex to show that Nup96 occurs in a spatial arrangement of two rings of 8 units with two Nup96 copies per unit giving a total of 32 Nup96 copies per pore. We use Artificial Intelligence assisted modeling in Alphafold to extend the existing cryo-EM model of Nup96 to accurately pinpoint the positions of the fluorescent labels and show the accuracy of the match between fluorescent and cryo-EM data to be better than 3 nm in-plane and 5 nm out-of-plane.

## Introduction

The Nuclear Pore Complex (NPC) is an essential molecular machine embedded in the nuclear envelope connecting the nucleus to the cytoplasm^1–3^. The NPC acts as a diffusion barrier that separates the nuclear compartment from the cytoplasm and works as a gateway for gene regulation^4,5^. It is indispensable in eukaryotic cellular processes such as regulating the transport of proteins and ribonucleoprotein^4,6,7^. The structure and molecular composition of the NPC, in particular the scaffold, has been extensively studied. Previous cryogenic electron microscopy (cryo-EM) and cryo-electron tomography (cryo-ET) studies have resolved the structure of the NPC scaffold to high-resolution^1–3,8–11^. The scaffold is composed of multiple copies of about 34 different nucleoporins (Nups) and these Nups are organized in three rings^12–14^. Previous studies have shown that the cytoplasmic ring (CR) and the nuclear ring (NR) are composed of multiple so-called Y-complexes and that Nup96 proteins are contained in these Y-complexes^10,15^. Each ring contains 16 Nup96 molecules, organised into units with eight-fold rotational symmetry^15–17^.

Super-resolution microscopy is emerging as a complementary technique for the study of biological structure, as it provides ‘diffraction-unlimited’ resolution^18–20^. Single molecule localization microscopy (SMLM) is one of these super-resolution techniques and obtains super-resolved images with a resolution of 10–20 nm by localizing single fluorescent emitters^18,21^. If many chemically identical structures, called particles, can be imaged, they can be registered and combined into one ‘super-particle’. With this strategy, the often poor degree of labelling of each individual particle can be mitigated and reconstructions with even better resolution than the typical 20 nm can be obtained^22–25^. Different 2D template-based particle fusion methods have been applied in SMLM to demonstrate the eight-fold rotational symmetry of the NPC^22,26,27^. These methods, however, carry the risk to generate reconstructions with a bias toward the template. Later, a template-free 2D registration approach could reveal the eight-fold symmetry of the NPC in an unbiased manner^28^. This template-free method was extended to 3D and used to reconstruct the 3D structure of Nup107 and Nup96 revealing two phase shifted rings with eight blobs per ring^29^. Yet, this 3D approach suffered from the ‘hot spot’ artefact, which could only be mitigated by applying prior knowledge about the eight-fold symmetry in a post-processing step. In another 3D particle fusion approach a model is fitted to the individual NPCs^30^ and the particles are combined in order of their similarity into the super-particle. This approach also shows two rings with each eight blobs or clusters in the NPC reconstruction, as expected, but interestingly some of the blobs are elongated and tilted in the plane of the rings. Limited by their high computational cost, neither the approach by Ref.^29^ nor by^30^ can reconstruct thousands of particles in a reasonable time. We recently introduced a template free and fast particle fusion approach^31^ that overcomes the limitation to computation speed, so that datasets of several thousands of particles (or more) are now accessible for structural analysis. In our iterative fast particle fusion approach^31^, we rotate and translate all the particles to fit a Gaussian Mixtures Model (GMM) using the Joint Registration of Multiple Point Clouds (JRMPC) method^32^, and subsequently update the positions and widths of the Gaussian centers. In each iteration round both the particles and the GMM are updated. Due to the inherent limitations of the joint registration method^32^, we can only obtain a locally optimal alignment of the particles which consist of several distinct clusters with different poses. We then classify^33^ these aligned particles with different poses and recombine them to obtain a globally optimal solution consisting of a single well-aligned structure.

Up to now SMLM of the NPC was able to reveal the eighth-fold rotational symmetry, resolve eight spots individually per ring and could show the separation of the nuclear and cytoplasmic rings in 3D. Although from cryo-EM work^15^ it is known that e.g. Nup96s occur as eight distinct units per ring, where a single unit contains two Nup96 copies, this could so far not be resolved by conventional SMLM. Here we show that each of the eight blobs indeed contains two fluorophores attached via a SNAP tag to the Nup96 unit by combining our fast template free particle averaging method^31^ with careful data analysis on 5 datasets (originating from five nuclei of five cells) of in total 4538 NPCs^30^. We made a detailed comparison of the outcome of our analysis to the cryo-EM data. To this end, we extended the incomplete Nup96 model derived from von Appen et al.^15^ by Alphafold^34^ to find the positions where the fluorescent SNAP-tags are expected to attach to the Nup96. Next, we registered our estimated positions of the fluorescent units to these expected positions of the SNAP-tag from the cryo-EM model and found the average distance between the SNAP positions derived from the cryo-EM model and from our SMLM data analysis to be < 3 nm laterally and 5 nm axially.

## Results

We have applied the methodological steps outlined in the “Methods” section to five Nup96 datasets. Dataset 1 was described earlier^30^; datasets 2–5 result from the same cell line and staining and imaging protocol. The number of analyzed NPCs per dataset are 368, 568, 706, 1178 and 1718 for a total of 4538 NPCs. Since the fast particle fusion method^31^ uses randomly generated GMM and leads to different reconstructions for the same dataset, we performed the fusion process 10 times for each dataset (including the combined dataset), resulting in a total of 60 reconstructions. Then we conduct data analysis carefully on these reconstructions to obtain our results.

### Particle fusion indicates inclined elliptical blobs

**Figure 1:**
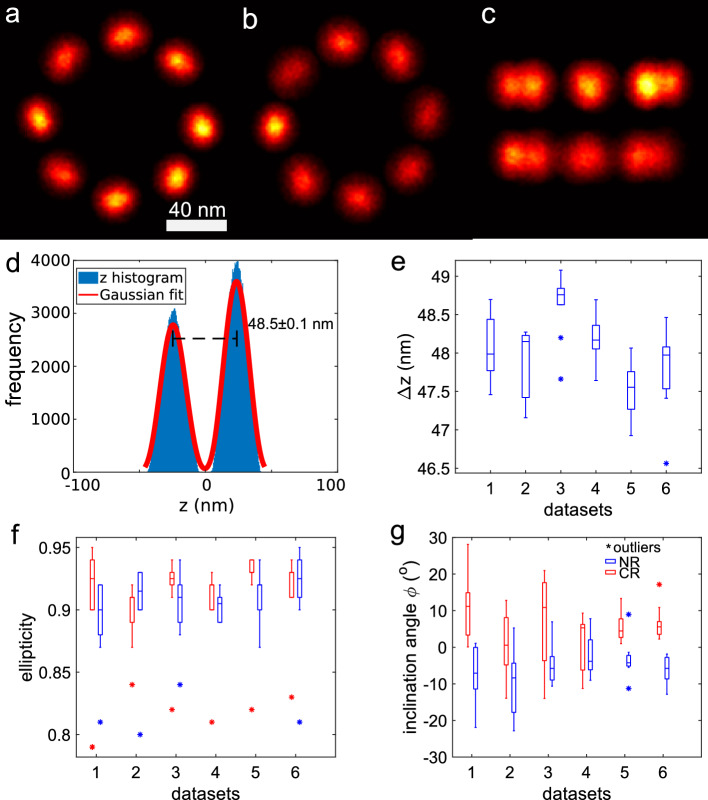
(**a**–**c**) One of the 3D reconstructions of Nup96 from 4538 NPC particles of the combined dataset. The output reconstruction includes all input particles. The reconstruction resolves two rings and 8 blobs per ring. (**a**) top view of the nuclear ring (NR), (**b**) top view of the cytoplasmic ring (CR), (**c**) Side view, (**d**) histogram of *z* coordinates of localizations in the reconstruction obtained from the combined dataset, and bimodal Gaussian fit to the data. The distance between the two peaks of the histogram is 48.5±0.1 nm. (**e**) Distance between the NR and CR for all the datasets. (**f**) Average ellipticity *e* of the elliptical blobs for the NR (blue) and CR (red), (**g**) average angle $\phi$ of the long axis of the elliptical blobs with the tangent to the ring in the *xy*-plane for the NR (blue) and CR (red) (negative angles indicate clockwise rotation). Scale bar in (**a**) is 40 nm and applies to (**b**,**c**) as well.

In Fig. 1a–c) we show one of the super-particle reconstructions of the combined dataset consisting of 4538 NPCs. In the top-view of the nuclear (NR) and cytoplasmic (CR) ring (a, b) the elongation of the blobs is clearly visible. Figure 1d) shows a histogram of the z-coordinates of the reconstruction in Fig. 1a–c). The localizations of the NR and CR are visible in the two peaks with a distance of 48.5 nm. Figure 1e) shows the average distance between the NR and CR *z*-histogram peaks for the 5 individual datasets and the combined dataset. The box plots give an indication of the statistical distribution of the fitted parameters for the 10 independent reconstructions that were made for each of the 6 datasets. The lower whisker of the plot extends to the minimum value of 46.6 nm, while the upper whisker extends to the maximum value of 49.1 nm. The median of the data is approximately 48.1 nm. In Fig. 1e–g), outliers are denoted by the asterisk (*) symbol.

Next, we analyzed the shape of the 16 separate blobs in the reconstructions. To that end, we first applied a routine for removing outlier localizations (see “Methods”), reducing the number of localizations by 32%. In Fig. 1f), the median value for the ellipticity is consistent, ranging from around 0.90 to 0.93 for all datasets. There are a few outliers ranging from 0.75 to 0.85, which are lower than the median values. Furthermore, it appears that the long axes of the ellipses make a median angle of around $\pm 5.5^{\circ }$ with the tangent to the rings, with an opposite sign for the CR and NR (Fig. 1g). If the ellipticity was due to a registration error or artefact of the particle fusion procedure, then the long axis of the ellipses would be aligned to the tangent of the rings. We can therefore conclude that the non-zero inclination of the elliptical blobs is evidence of a structural feature. The most simple explanation is that each elliptical blob is the composite of localization events of emitters that bind to two distinct binding sites, i.e. the particle fusion directly suggests that Nup96 occurs in an arrangement with two Nup96 copies per unit. We note that the evidence for (at least) two binding sites comes from interpreting the structural feature of ellipticity. The binding sites cannot be directly observed as two distinct spots in the reconstructions. It is noticeable that the blobs in the NR have a better signal-to-noise ratio (SNR) than the blobs in the CR (Fig. 1c) and the NR has a significantly higher number of localizations compared to the CR, with around 22% more localizations (Fig. 1d). This difference in quality may be caused by the way the JRMPC method fuses the data. In a global optimization, a better registration may be achieved for one part of the structure (here NR) at the expense of the registration quality of another part of the structure (here CR). Additionally, structural heterogeneity could also contribute to the observed differences in registration quality.

### Unconstrained and symmetry constrained fitting of binding sites

We investigated two approaches to estimate the positions of the two binding sites in the Nup96 units. Firstly, we used unconstrained fitting of 16 anisotropic Gaussian centers for the NR and CR separately, and secondly we used constrained fitting in which we impose the eight-fold rotational symmetry [according to Eqs. (1) and (2)].

**Figure 2:**
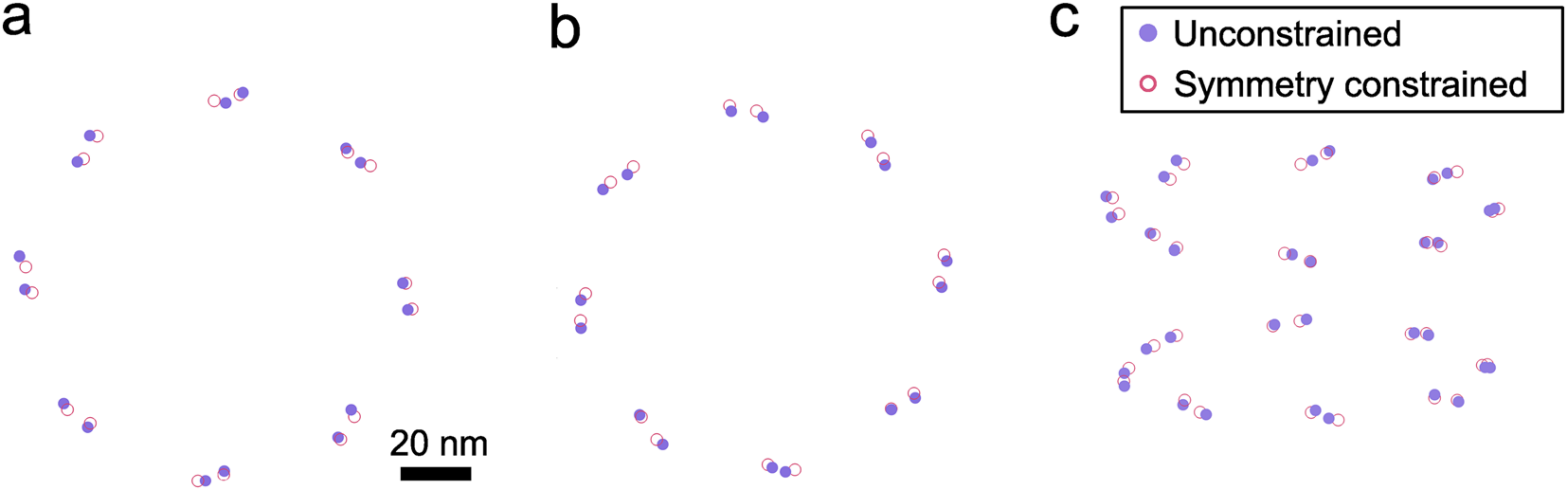
Locations of binding sites obtained from an unconstrained fit (purple) and from an eight-fold symmetry constrained fit (pink). These fits are obtained from the reconstruction displayed in Fig. 1a–c) of the combined dataset. Top view of binding sites for the NR (**a**) and CR (**b**). (**c**) Oblique view of CR and NR for projection at an angle $15^\circ$ with the *xy*-plane. Scale bar in (**a**) applies to (**b**,**c**) as well.

We found the distance between the 16 binding sites of the two fitting approaches for the combined data to be $2.8\pm 1.7$ nm for the NR and $3.6\pm 1.4$ nm for the CR (see Fig. 2). This small difference indicates that our data matches well with the eight-fold symmetry and that the use of the symmetry constraint in the fit is a valid procedure.

### Structural parameters of the Nup96 unit

**Figure 3:**
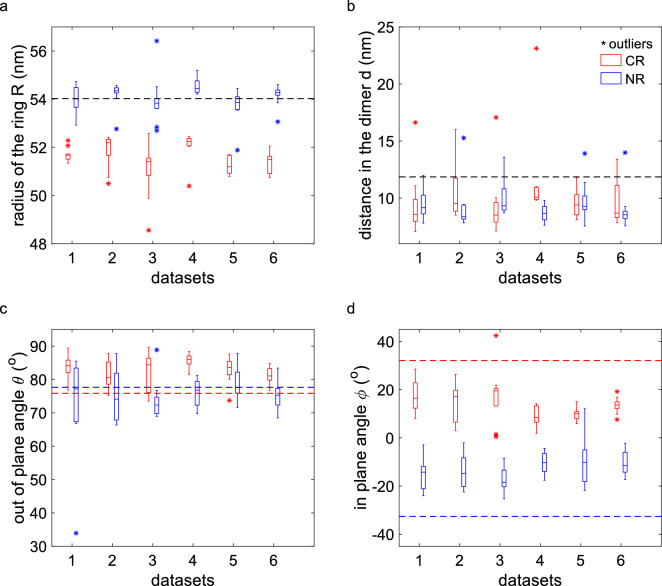
Structural parameters for eight-fold symmetry constrained fitting for the NR (blue) and CR (red) for all datasets. Parameters of fluorophore locations derived from the cryo-EM model are shown as dashed lines and the outliers identified from SMLM results are denoted by the asterisk (*) symbol. (**a**) Radius for the NR and CR, the NR radius is around 54.1 nm and the CR radius is around 51.6 nm. The reference radius is 54.2 nm. (**b**) Distance between the binding sites in the units. The distance in the units is $9.7\pm 2.2$ nm and the reference value is 11.8 nm. (**c**) Out-of-plane angle $\theta$ of the line connecting the two emitters in a unit to the *z*-axis, $\theta = 79.1^\circ \pm 7.3^\circ$ and the reference value is $\theta =76.8^\circ$. (**d**) In-plane angle $\phi$ of the line connecting the two emitters in the unit to the tangent of the ring in the *xy*-plane, $\phi \sim -12.8\pm 7.0^\circ$ for the NR and $\phi \sim +13.7\pm 7.5^\circ$ for the CR. The reference angle is $-32.6^\circ$ for the NR (blue dashed line) and $32.1^\circ$ for the CR (red dashed line). For panels (**c**,**d**), positive angles indicate counterclockwise rotation.

Figure 3 shows the structural parameters (radius *R* of CR and NR, distance *d* between the two binding sites in the unit, out-of-plane tilt angle $\theta$ of the unit, in-plane inclination angle $\phi$ of the unit) for all datasets for the case of symmetry constrained fitting. For comparison we also show the reference values from the cryo-EM data ($R=54$ nm, $d=11.8$ nm, $\phi =-32.6^{\circ }$ (NR), $\phi =32.1^{\circ }$ (CR), $\theta =76.8^{\circ }$)^15,34^. The structural parameters obtained from the symmetry constrained fitting are relatively consistent between the different reconstructions and close to the cryo-EM reference values. In particular the radius and distance between the binding sites in the unit match well, where the angles are a bit more off compared to the cryo-EM model. There are some variations between the datasets, where datasets 3 and 4 appear to have more scattered localizations than the others resulting in visually poorer reconstructions for the NR and CR. The estimated uncertainties of the parameters for the NR are smaller than for the CR, consistent with the lower reconstruction quality of the CR compared to the NR. The radius of the NR matches very well with the cryo-EM reference value, whereas the radius of the CR is consistently about 2.5 nm smaller. The estimate of the distance between the binding sites in the unit is somewhat smaller than the cryo-EM reference distance. We find that the in-plane inclination angle has the opposite sign for the NR and the CR, which is consistent with the cryo-EM model. The estimated magnitude of the in-plane inclination angle is somewhat smaller than the cryo-EM reference values. A larger quantitative mismatch is found for the out-of-plane tilt angle, which we attribute to the localization uncertainty in the axial direction, which is two to three times larger than the uncertainty in the *xy*-plane^35^.

### Comparison of cryo-EM and SMLM

**Figure 4:**
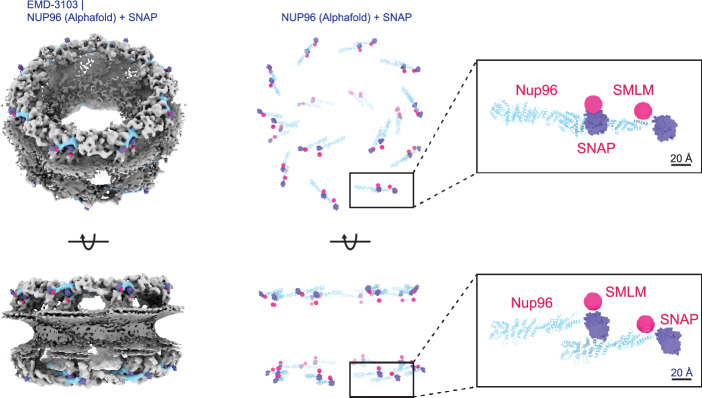
Overlay of the fluorophore positions from the SMLM particle fusion data (pink) and the SNAP-tag derived from the cryo-EM data (purple). For our overall SMLM emitters (pink), the lateral distance between a unit are 9.1 nm for NR and 10.0 nm for CR. The axial distances between a unit are 2.4 nm for NR and 1.2 nm for CR. The SNAP tags (purple) have lateral distances between a unit of 11.6 nm for NR and 11.5 nm for CR as well as axial distances of 2.5 nm for NR and 2.9 nm for CR.

Figure 4 shows a direct comparison between the positions of the SNAP tags predicted from the cryo-EM data, and the positions of the emitters according to the SMLM particle fusion data. The emitters of SMLM are generated from the overall symmetry constrained fit parameters obtained from averaging all 60 fits. We measured the distance between the corresponding emitters of our symmetry constrained fit and SNAP tags predicted from the cryo-EM. For the NR we find in-plane distances of 2.1 nm and 2.3 nm, and out-of-plane distances of 5.1 nm and 5.0 nm for the two emitter positions in the unit, for the CR we find in-plane distances of 4.2 nm and 0.4 nm, and out-of-plane distances of − 3.5 nm and − 5.2 nm for the two emitter positions in the unit. Please note that we cannot assign the fluorophore position directly in our structural model, which is limited to modeling the SNAP tag position relative to NUP96, whereas we measure the fluorophore position directly in SMLM. The expected distance between the SNAP-tag and the fluorophore position is 1–2 nm. Overall the agreement in the plane of the NPC rings is rather well, with an overall error of just $2.2\pm 1.4$ nm.

The lateral error also seems free of a systematic bias between the cryo-EM based and the SMLM particle fusion based position estimates. This stands in contrast to the axial position estimates where we find a distance between the top emitters in the CR and the NR of 48.4 nm, and between the bottom emitters in the CR and NR of 47.3 nm. It appears that the SMLM data give a distance between the NR and CR that is systematically smaller than the distance obtained from the cryo-EM model, with an average bias in estimated NPC thickness obtained from the cryo-EM/SMLM comparison of 9.4 nm. Figure 1e) shows the estimated distance between the NR and CR for all datasets as well as Fig. 1d) displays the histogram of the *z* coordinates of the 244,946 localizations in the reconstruction obtained from the combined dataset. We find the distance between the NR and CR in SMLM to be $48.0\pm 0.4$ nm, while the distance between the rings in the EM model is $57.2\pm 0.2$ nm, a bias of about 9.2 nm. This analysis of the underlying axial localization data suggests that the found bias is not due to an artefact of the particle fusion method.

## Discussion

Overall, our analysis of the SMLM particle fusion points to a Nup96 unit structure that matches well with the cryo-EM data. Compared to the cryo-EM model, however, one major inconsistency remains. Namely, the height of the NPC is estimated from SMLM particle fusion to be about 9 nm less than that from cryo-EM. Similar ring separation values for Nup96 have been reported in other SMLM studies^36,37^, and have a larger deviation from the cryo-EM model than the levels of statistical error. There are a number of factors that could contribute to this discrepancy. Firstly, the differences in cell lines and the functional state of cells may play a role, as discrepancies in the height of NPCs have previously been observed in cryo-ET structures from different cell lines, with a $\sim$ 5 nm height difference observed between in situ NPC observed in HeLa and HEK cells. These differences could correspond to distinct functional states involving e.g. dilation of the inner ring^3,9^. Secondly, the axial localization data is calibrated based on reference images of NPCs that are oriented sideways. In a side view the two rings are clearly separated in the image plane. There the localization precision is much better ($2-3\times$) than in the axial direction and the separation can be estimated easily and used to calibrate the axial coordinate^37^. The side views are obtained from NPCs at the sides of the nucleus, while the other NPCs are located in the nuclear envelope that lies flat against the cover slip. Thirdly, if the fluorsophore motion is (partly) restricted during imaging, dipole orientation effects on single molecule imaging can have an impact on the lateral position estimation, resulting in biases on the order of $\sim$ 10 nm^38^. A confounding factor is the analysis of 3D SMLM data of Nup107, which is adjacent to Nup96 in the NPC scaffold, and which was found to have a ring separation of 60 nm in SMLM particle fusion^29^—in agreement with the cryo-EM data. It is worth mentioning that the Nup107 particles were obtained from another experiment in which the cell line and the functional state of the cell were different from the datasets analysed in this paper.

The estimated structural parameters for the NR appear to be more consistent between datasets than for the CR (see Fig. 3). We attribute this to the fact that the localizations of the CR are more scattered, and in turn the reconstructed super-particle is of lower quality there. The underlying reason could be that the 3D particle fusion favors the alignment of one ring, which leaves the other ring more blurred—in particular in the axial direction. A possible point of improvement could be to re-register the localizations in the CR by our particle fusion method separately, which in the end may give rise to a reconstruction of the CR with better quality.

Other technical improvements to the data analysis can also be envisioned. Firstly, the removal of outlier localizations is now done via a cascade of adding an extra Gaussian center to the GMM and subsequently filtering localizations according to the density of localizations. A single integrated approach may improve robustness of the data analysis procedure. Secondly, initial parameter settings could result in a better convergence to global optima of GMM fitting. A more principal refinement of the current analysis relates to model selection. The current analysis relies on the simplest model that can explain the observations, namely that the Nup96 appears in a unit structure of two copies. A possible improvement may therefore be found in a statistical criterion that supports that there are 32 emitters in the NPC, as opposed to another multiple of 16.

Recently, Helmerich et al.^39^ speculated that fluorophores at distances below about 10 nm cannot be reliably resolved with SMLM although the precision given by the microscope, data analysis pipeline, and observed single molecule photon count should allow such a distinction. Energy transfer between the close-by fluorophores is assumed to cause re-excitation of the emitters at the beginning of the experiment followed by bleaching of both, preventing observation of them individually as required for SMLM. In particular with the introduction of MINFLUX and related techniques^36,40–42^ that offer nanometer localization precision at low photon count these observations could frustrate direct imaging of the unit separation in the NPC except for rare cases and leave careful data analysis as presented here as the only remaining tool^43^. Gwosch et al.^44^ fused 162 NPC particles obtained from the MINFLUX data of^36^ by the all-to-all particle fusion method^29^. Their result does not depict the unit structure we demonstrate here. This may be due to a number of factors such as low density of labelling or few localizations per particle.

Very recently, a novel DNA-barcoding method, “Resolution Enhancement by Sequential Imaging” (RESI), has significantly improved the localization precision^45^. By applying the data-driven average method^30^ to give a fusion of 1217 particles, individual Nup96 proteins within a unit could be distinguished. The lateral and axial distance were 11.9 ± 1.2 nm and 5.4 ± 0.4 nm, respectively. These values are somewhat larger than the values obtained from our symmetry constrained fit in Fig. 4. We measured each blob’s long axis inclination angle and ellipticity from Fig. 2g of the RESI paper. We found for the CR that the inclination angle is $-9.3 \pm 3.1^{\circ }$, while for the NR the inclination angle is $5.9 \pm 1.6^{\circ }$. In comparison, Fig. 1g gives median inclination angles of ± 5.5$^{\circ }$ for the CR and NR blobs with an opposite sign. The seeming difference in handedness of the structure, i.e. the sign of the in-plane inclination angle, of the RESI results compared to our results and those of Ref.^30^ is due to a difference in projection (viewed from the CR side vs. viewed from the NR side). The average ellipticity for the CR and NR blobs in the RESI result is 0.86 ± 0.02 and 0.88 ± 0.03, respectively. We found from Fig. 1f, ellipticity ranges from 0.90 to 0.93 for the CR and NR blobs, respectively, in good agreement. Overall, the conclusions on the unit structure are similar, differences may be attributed to biological variations and differences in imaging conditions.

In conclusion, we have demonstrated that SMLM can reveal 3D structures in the small nanometer range. Our high-resolution particle fusion reconstructions and subsequent data analysis enabled the precise estimation of the positions of 32 emitter binding sites for Nup96 in the NPC. The comparison with cryo-EM data shows consistency better than 2.5 nm laterally, but also a bias in the NPC height estimation of about 9.2 nm. The latter inconsistency is poorly understood, and requires further study by the research community.

## Methods

### Particle averaging

We applied our previously published particle averaging method^31^ to five NPC SMLM datasets individually and all 5 combined (total of 4538 NPCs). Dataset 1 with 368 NPCs was previously described^30^ and the other datasets were obtained under the same experimental conditions. Each dataset corresponds to a single cell. Our fast particle fusion method first jointly aligns the input particles with a randomly generated Gaussian Mixture Model (GMM). The number of Gaussian components and the initial Gaussian standard deviation of the GMM are pre-set for better convergence in the iterative optimisation step^32^. Since the joint registration can be trapped at a local optimum, which contains several groups of particles (clusters) with different overlapping poses in the reconstruction, we apply an unsupervised classification method^33^ to separate these clusters and reconnect the cluster to the same pose. We use the default values given in^31^ for the data fusion algorithm, except for the number of Gaussian components *K* in the Gaussian Mixture Model (GMM) and the initial Gaussian standard deviation of the Gaussian components. We set $K=34$, that is 32 Gaussian components for 32 binding sites and 2 to accommodate false positive localizations of the nuclear ring and the cytoplasmic ring. The initial poses of the NPC particles (long axis of the cylinder) are roughly aligned with the optical axis, as the nuclear membrane runs along the cover slip. We can use this to set an initial Gaussian standard deviation to  33 nm, smaller than the overall size of the NPC, to make the algorithm converge faster. We performed particle averaging 10 times on each dataset and thus obtained a total of 60 super-particles to incorporate the statistical variability induced by the randomly generated GMM into the analysis. All super-particles, shown in the Appendix, consistently exhibit two rings, each containing 8 elliptically shaped blobs across all 5 datasets as well as for the combined dataset.

### Splitting in NR and CR

We obtain super-particles from the particle averaging of the different datasets that have the same shape but different absolute poses in 3D space. For better comparison and easier analysis we align their global pose such that the rings are perpendicular to the *z*-axis of our global coordinate system. We do so by registering the super-particles to a fixed template which has two rings and each ring contains eight points with eight-fold rotational symmetry. The center of the template is located at the origin of the coordinate system. The distance between the two rings in the template is 50 nm and the radius of each ring is 55 nm. Finally, we rotate the super-particles around the *x* and *y*-axis from $-3^\circ$ to $3^\circ$ in steps of $0.2^\circ$ to find the minimum full width at half maximum (FWMH) of the histogram of *z*-coordinates. Then, we split the super-particle into two 8-blob rings according to their *z*-coordinates for further analysis. These rings represent the nuclear (NR) and cytoplasmic (CR) ring of the NPC.

### Outlier localization filtering

Next, we remove outlier localizations in each ring. To this end, we use all the localizations in a ring and fit a randomly initialized GMM with 9 Gaussian components using again the JRMPC method. In the *xy* plane all the GMM centers are randomly generated inside a 2D box with a diameter of 100 nm. The z-coordinates of these initial Gaussian centers are set to 0. The standard deviations of the initial Gaussian components are set to 33 nm. Thus, an initial GMM with all Gaussian components locating nearby the localizations and having comparable standard deviations to the size of the ring is suitable for JRMPC to converge. In all cases we find that there are 8 components with similar (small) standard deviations and one component with a large standard deviation, indicating the outlier component. We remove the localizations in the latter component from the data (this is about 5% of the number of localizations). As the automated data analysis is sensitive to outlier localizations, we apply further outlier filtering based on the density of localizations to the blobs. We remove all localizations with a local density smaller than a threshold such that every blob is separated clearly from its adjacent blobs in the reconstruction. We experimented with various density thresholds and determined that a value of 95% of the mean density of a single blob is suitable for all reconstructions. This latter operation removes about 1/3 of the localizations.

### Ellipticity measurement in per blob

The long axes of the elliptically shaped blobs in a ring are tilted with respect to the circumference of the ring. To investigate this further, we measure the ellipticity of each projected blob onto the *xy*-plane. The ellipticity is defined as $e=b/a$, where *a* and *b* are the lengths of the long and short axis, respectively. We also measure the inclination angle between the long axis of each blob and the tangent to the overall 2D ring. We then calculate the distribution of the ellipticity and inclination angle over the set of values for the 8 blobs of the 10 reconstructions in each ring.

### Anisotropic Gaussian mixture fitting to 8 blobs per ring

The inclination of the elliptical shape of the blobs with respect to the rings indicates that more than one emitter is present per blob. As moreover the inclination angle is also opposite in sign for the NR and CR, the inclination is not a reconstruction artifact. The unit of two Nup96, however, is not resolvable due to the limited localization precision, residual drift or registration error. We assume that there are two SNAP emitters per blob based on the prior knowledge from the cryo-EM model that there are two Nup96 copies per blob. We again use a GMM with 16 components to fit the 8 blobs per ring and interpret the Gaussian components’ centers as the positions of the emitters. For the fitting procedure we use the following heuristics: The initial standard deviation for the Gaussian fitting is equal in the *x* and *y* direction (in-plane), but two times larger in the *z*-direction (which is aligned with the optical axis), because the axial localization uncertainty is typically two to three times larger than in the *xy*-plane^35^. Furthermore, we assume that all Nup96 units in a ring are identical, i.e. the Gaussian components in the GMM have identical diagonal covariance matrices.

We use iterative Expectation-Maximization (EM) to find the optimal GMM by maximizing the likelihood for the GMM to fit the localizations^46^. The shared diagonal covariance matrices, centers of Gaussian components and posterior probabilities of component memberships are the fitting parameters and updated iteratively. The obtained Gaussian centers from fitting 16 anisotropic Gaussian mixture model are represented by $G=\{\textbf{g}_i\}_{i=1}^{16}$ with ${\textbf{g}_i}=(g_{ix},g_{iy}, g_{iz})$.

### Incorporation of the eight-fold symmetry into the fitting

**Figure 5:**
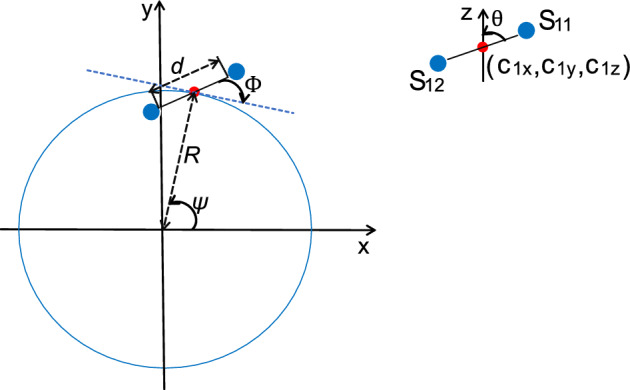
Schematic for structural parameters of the 8 pairs of two binding sites with overall eight-fold symmetry. The red point is the center of the first pair and the blue points are the binding sites in the units.

We have employed a second fitting method to make use of the eight-fold symmetry, as unconstrained anisotropic GMM fitting is sensitive to the setting of the initial Gaussian centers. To this end we generate 16 points with eight-fold symmetry as the initial centers of the anisotropic GMM fitting for every ring (see Fig. 5). The 16 binding sites defined by the point set $S=\{\{\textbf{s}_{kj}\}_{j=1}^{2}\}_{k=1}^{8}$ are characterized by 6 parameters: ($c_{1x}$, $c_{1y}$, $c_{1z}$, *d*, $\theta$, $\phi$), where ${\textbf{c}_1}=\left( c_{1x}, c_{1y}, c_{1z}\right)$ is the center of the first ($k=1$) unit, *d* is the distance between the two binding sites in the unit, $\theta$ is the angle between the line connecting the two binding sites in a unit and the positive *z*-axis ($0\le \theta \le \pi$), and $\phi$ is the angle between the projection of this line on the *xy*-plane to the tangent of the ring in *xy*-plane ($0\le \phi \le 2\pi$). The in-plane coordinates of the unit center can be parameterized as ${\textbf{c}_1}=\left( c_{1x}, c_{1y}\right) =R\left( \cos \psi ,\sin \psi \right)$, with *R* the radius of the ring and $\psi$ the in-plane angle. The coordinates of the two binding sites in the first unit are thus given by:

1 $$\begin{aligned} {\textbf{s}_{11}}= & {} (R\cos \psi , R\sin \psi , c_{1z})+\frac{1}{2}d\left( -\sin \theta \sin \left( \phi +\psi \right) ,\sin \theta \cos \left( \phi +\psi \right) ,\cos \theta \right) , \end{aligned}$$

2 $$\begin{aligned} {\textbf{s}_{12}}= & {} (R\cos \psi , R\sin \psi , c_{1z})-\frac{1}{2}d\left( -\sin \theta \sin \left( \phi +\psi \right) ,\sin \theta \cos \left( \phi +\psi \right) ,\cos \theta \right) . \end{aligned}$$

We choose emitter 1 to be the emitter that lies above the center plane of the ring, i.e. we restrict the polar angle to $0\le \theta \le \pi /2$. The coordinates of the binding sites of the $k=2,\dots ,8$ other units can be derived by rotating these points by $(k-1)\pi /4$ in the *xy*-plane. The parameters for the initial GMM centers are (0, 53.5 nm, ± 24 nm, 13 nm, $\pi /2, \pi /4$), where $+$ corresponds to NR and − to CR. The in-plane angle $\phi$ is randomly generated in the interval 0 to $\pi$. We find the parameters of the symmetry constrained point sets *S* from the coordinates of the unconstrained point set *G* by minimizing the mean square error between the point sets *S* and *G* with the quasi-Newton method^47^ implemented in MATLAB.

### Comparison with the cryo-EM model

In order to relate the found emitter positions to available structural data on the human NPC^15^, we modeled the full-length Nup96 structure using Alphafold2^34^ and added the SNAP-tag at the carboxyl-terminal position on the Nup96s. We rigid-body fitted the full-length Nup96 into the cryo-EM density using residues 881-1817 from PDB ID 5a9q as anchor residues^15^. As expected, the SNAP tag protrudes from the NPC assembly, adding support for its correct placement. We then symmetry-expanded the fitted model to generate the full NPC assembly and denote the center-of-mass of the O$^{6}$-benzylguanine-AF647 (BG-AF647) to represent the emitter position.

To align the cryo-EM and SMLM models, we first measured the parameters of the SNAP tags from the cryo-EM model. Then, we regenerated the SNAP tags in the EM model and the overall eight-fold symmetric emitter positions in the SMLM reconstructions from their fit parameters individually, ensuring that they share the same center as the first unit. The predicted SNAP tag positions based on the cryo-EM model are represented by position vectors ${\textbf{m}_{kj}}=(m_{kj,x},m_{kj,y}, m_{kj,z})$ for $k=1,\ldots 8$ and $j=1,2$. Using this set of position vectors reference values for the radius *R* of the CR and NR, the distance *d* between the two binding sites in the unit, the in-plane inclination angle $\phi$ of the unit, and the out-of-plane tilt angle $\theta$ of the unit can be obtained from equations similar to Eqs. (1) and (2).

The two models can be directly compared by computing the in-plane and axial distance of the cryo-EM SNAP tag positions to the estimated emitter positions from SMLM particle fusion:

3 $$\begin{aligned} b_{xy,jk}= & {} \sqrt{\left( m_{kj,x}-s_{kj,x}\right) ^{2} + \left( m_{kj,y}-s_{kj,y}\right) ^{2}} \end{aligned}$$

4 $$\begin{aligned} b_{z,jk}= & {} m_{kj,z}-s_{kj,z}. \end{aligned}$$

As the cryo-EM data satisfies the eight-fold rotational symmetry by construction we only compare the cryo-EM SNAP tag positions to the estimated emitter positions from SMLM particle fusion that are obtained from the symmetry constrained unit fits. That means that there are only four distinct values for $b_{xy,jk}$ and four distinct values for $b_{z,jk}$ (CR and NR, two emitter positions per unit).

### Supplementary Information

Supplementary Information.

## Data Availability

The dataset and source codes for this work are publicly available at https://github.com/wexw/Particle-fusion-Nup96.git.
