# Publisher Correction: Particle fusion of super-resolution data reveals the unit structure of Nup96 in Nuclear Pore Complex

**DOI:** 10.1038/s41598-023-43079-w

**Published:** 2023-09-28

**Authors:** Wenxiu Wang, Arjen Jakobi, Yu‑Le Wu, Jonas Ries, Sjoerd Stallinga, Bernd Rieger

**Affiliations:** 1 Faculty of Applied Sciences, Delft University of Technology, Delft, The Netherlands; 2 Cell Biology and Biophysics Unit, European Molecular Biology Laboratory (EMBL), Heidelberg, Germany; 3 Department of Chromosome Biology, University of Vienna, Max-Perutz LabsCenter for Molecular Biology, Vienna, Austria

Correction to: *Scientific Reports* 10.1038/s41598-023-39829-5, published online 16 August 2023

The original version of this Article contained an error in the upper inset of Figure 4, where the atomic model was missing. The original Figure 4 and accompanying legend appear below.

**Figure 4:**
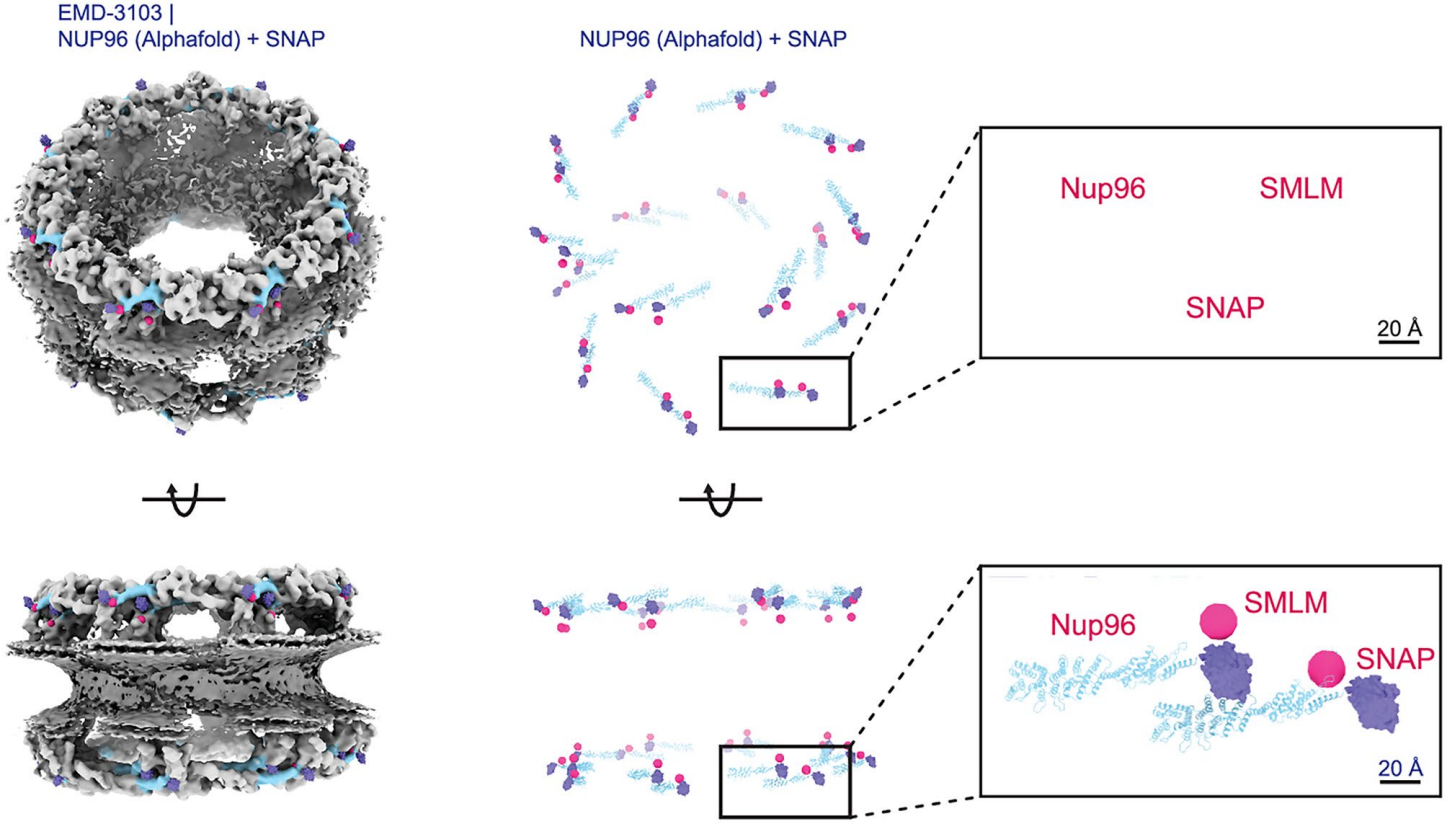
Overlay of the fluorophore positions from the SMLM particle fusion data (pink) and the SNAP-tag derived from the cryo-EM data (purple). For our overall SMLM emitters (pink), the lateral distance between a unit are 9.1 nm for NR and 10.0 nm for CR. The axial distances between a unit are 2.4 nm for NR and 1.2 nm for CR. The SNAP tags (purple) have lateral distances between a unit of 11.6 nm for NR and 11.5 nm for CR as well as axial distances of 2.5 nm for NR and 2.9 nm for CR.

The original Article has been corrected.

