# Supplementary material for: Particle fusion of super-resolution data reveals the unit structure of Nup96 in Nuclear Pore Complex
Source: Sci Rep. 2023 Aug 16;13:13327. doi: 10.1038/s41598-023-39829-5 (PMC10432550; doi:10.1038/s41598-023-39829-5)
Supplement: Supplementary file 1 — Supplementary Information. [file 41598_2023_39829_MOESM1_ESM.pdf]

PARTICLE FUSION OF SUPER-RESOLUTION DATA REVEALS THE UNIT STRUCTURE OF NUP96 IN NUCLEAR PORE COMPLEX

Wenxiu Wang [1], Arjen Jakobi [1], Yu-Le Wu [2], Jonas Ries [3], Sjoerd Stallinga [1,] and Bernd Rieger [1,*]*

[1]Faucalty of Applied Sciences, Delft University of Technology, Delft, The Netherlands
[2]Cell Biology and Biophysics Unit, European Molecular Biology Laboratory (EMBL), Heidelberg, Germany
[3]University of Vienna, Max-Perutz Labs, Center for Molecular Biology,
Department of Chromosome Biology, Vienna, Austria

Appendices

A. DATA FUSION RESULTS WITHOUT FILTERING OUTLIER LOCALIZATIONS

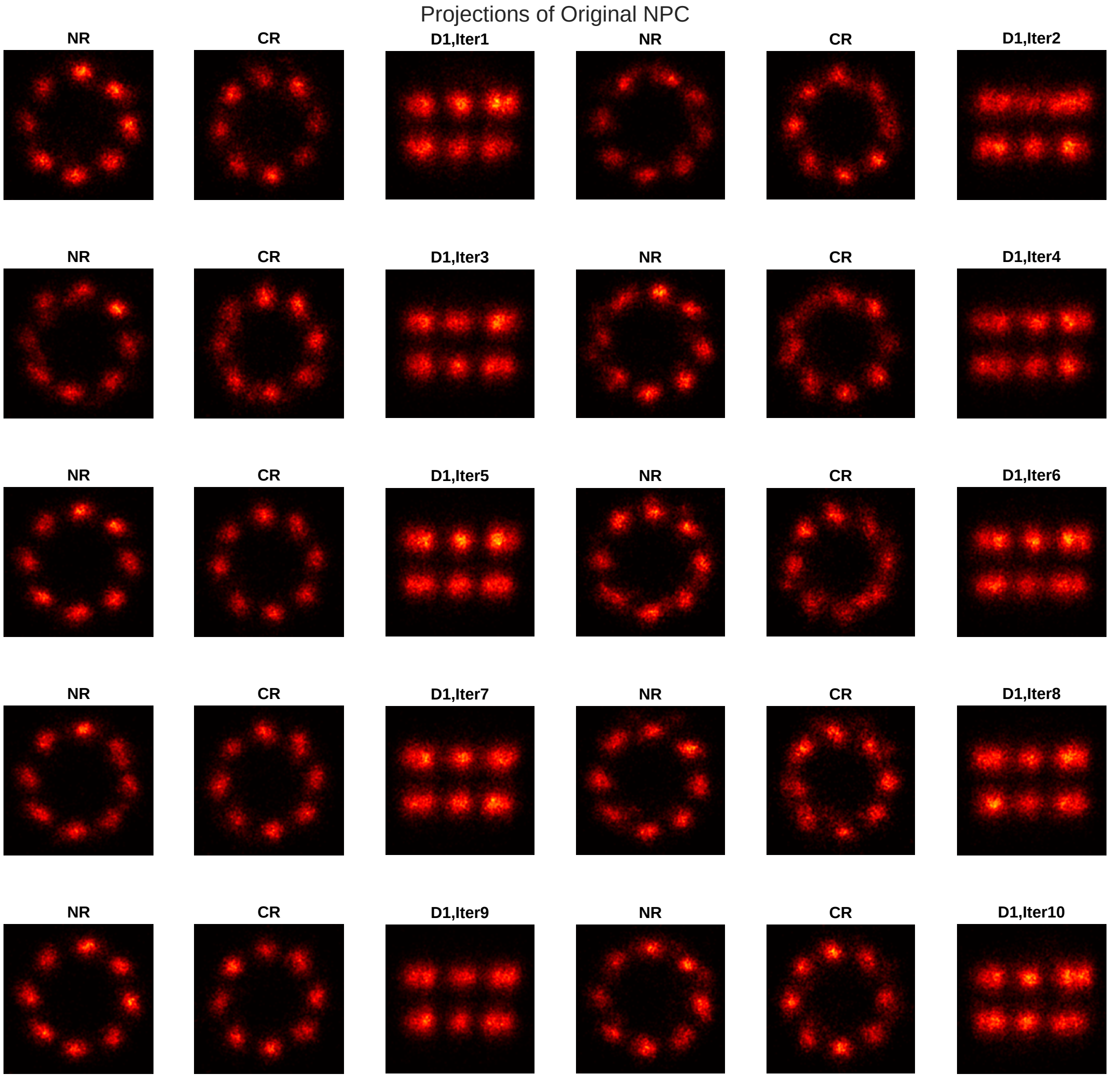

Fig. S1. Data fusion results for dataset 1 for 10 randomly different GMM initializations without filtering outlier localizations.

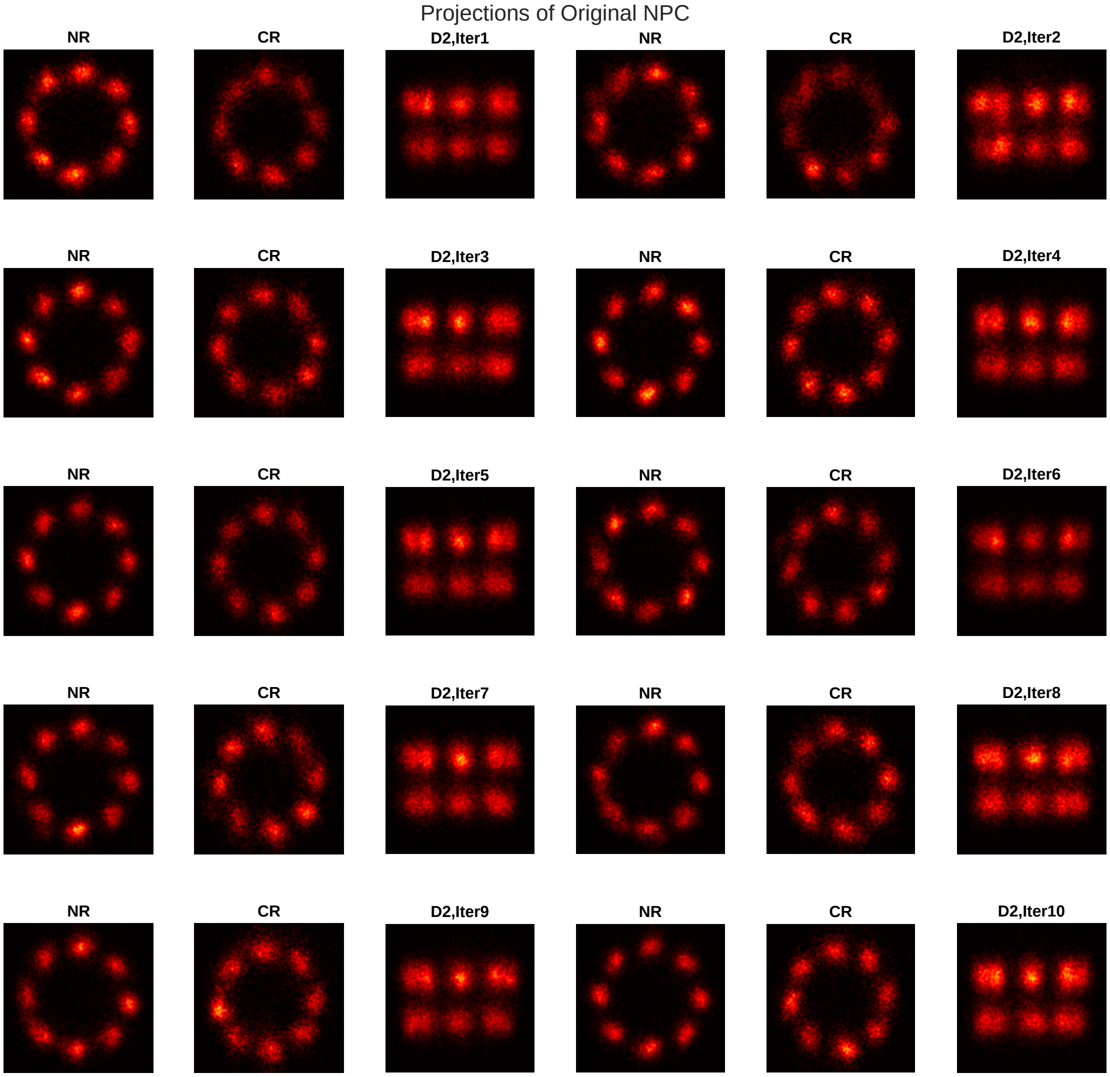

Fig. S2. Data fusion results for dataset 2 for 10 randomly different GMM initializations without filtering outlier localizations.

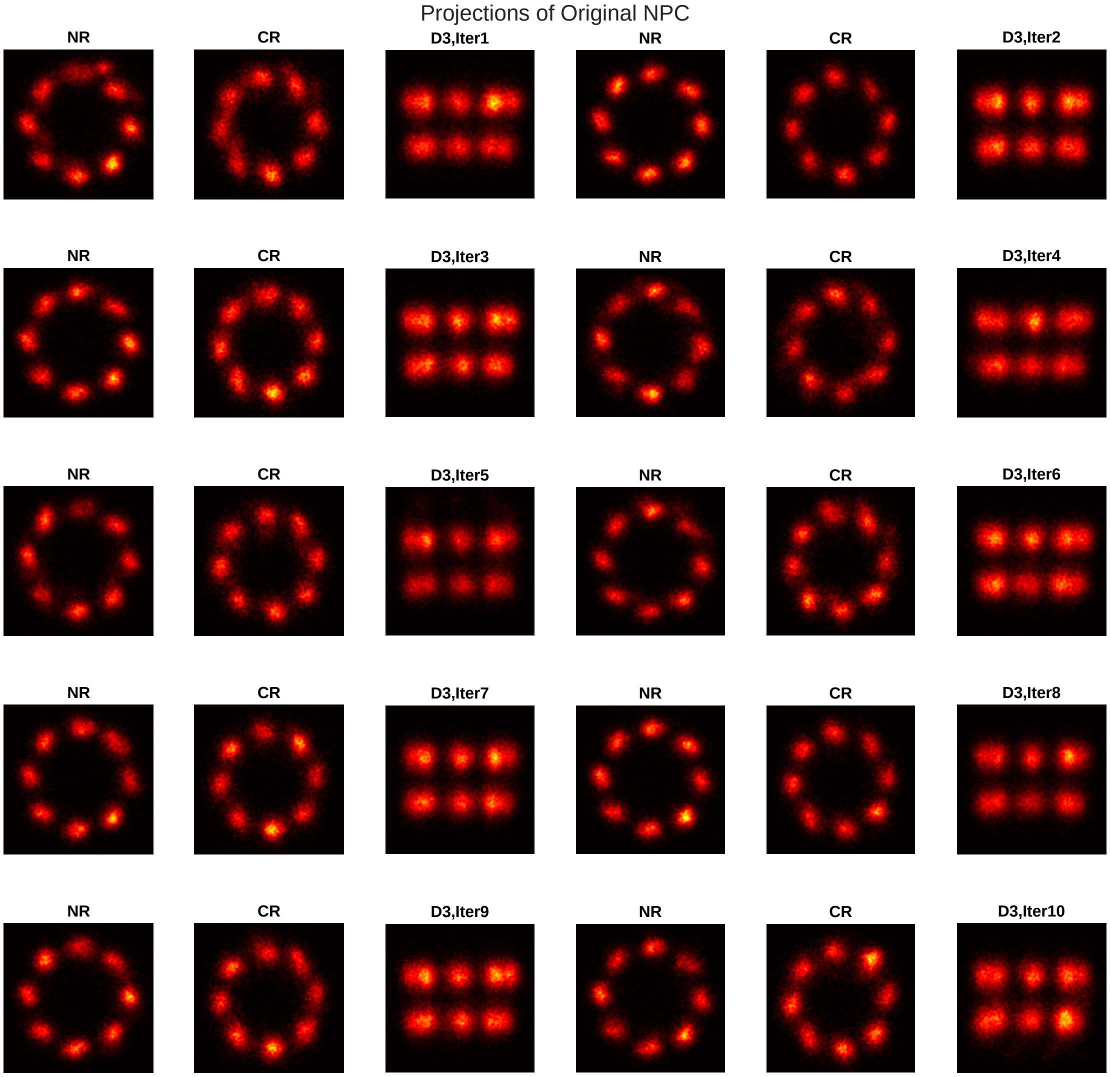

Fig. S3. Data Fusion results for dataset 3 for 10 randomly different GMM initializations without filtering outlier localizations.

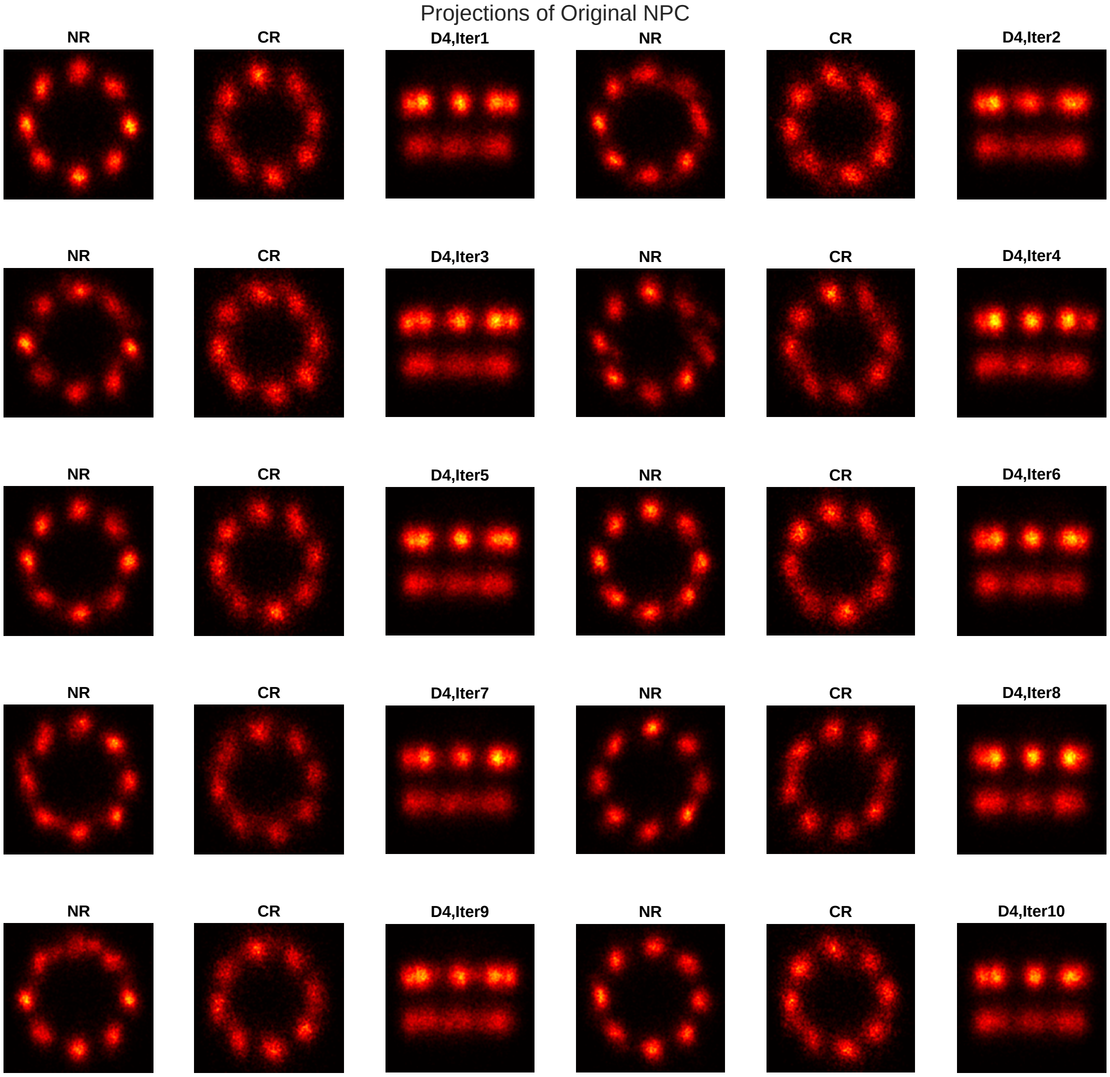

Fig. S4. Data Fusion results for dataset 4 for 10 randomly different GMM initializations without filtering outlier localizations.

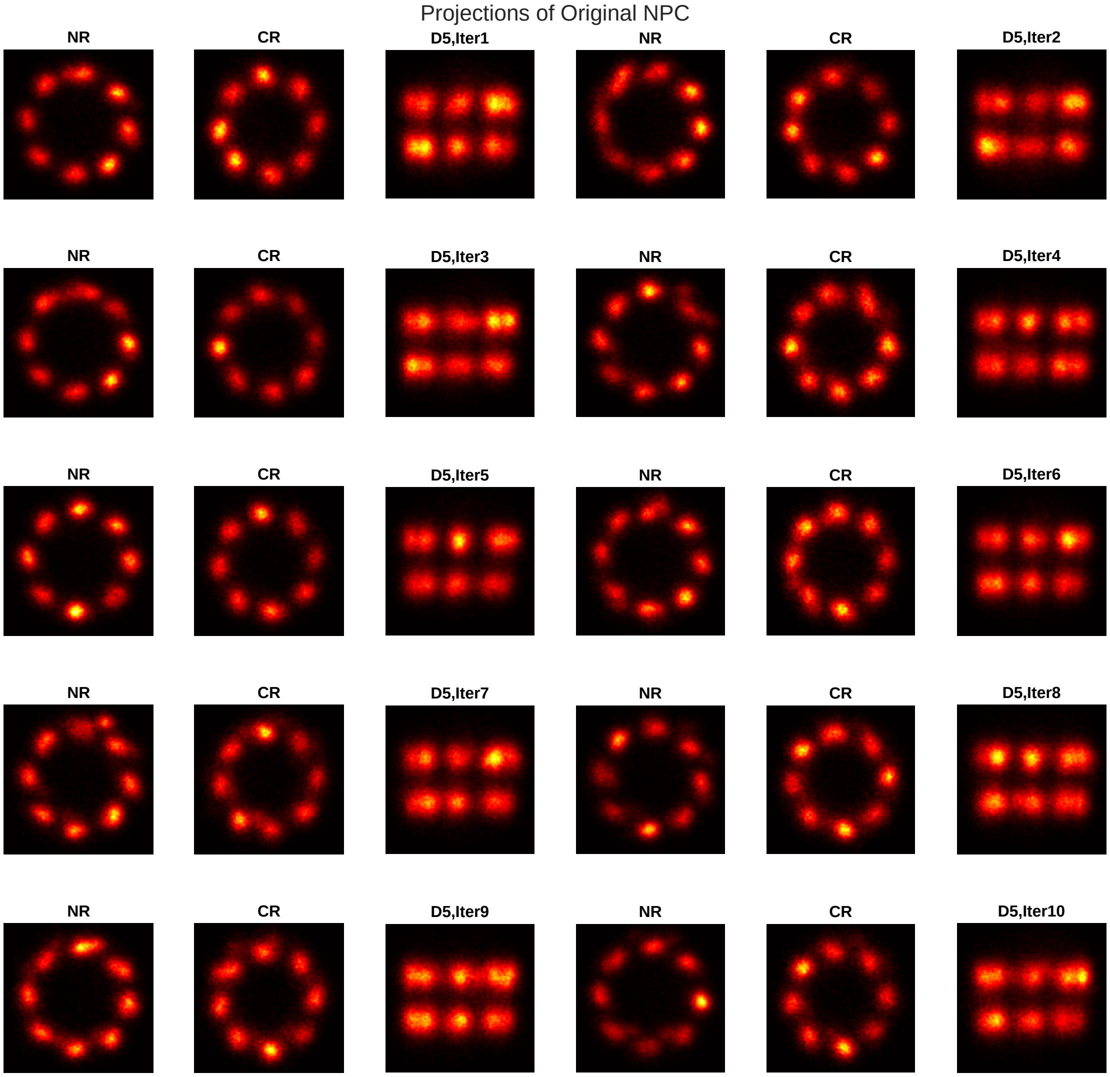

Fig. S5. Data Fusion results for dataset 5 for 10 randomly different GMM initializations without filtering outlier localizations.

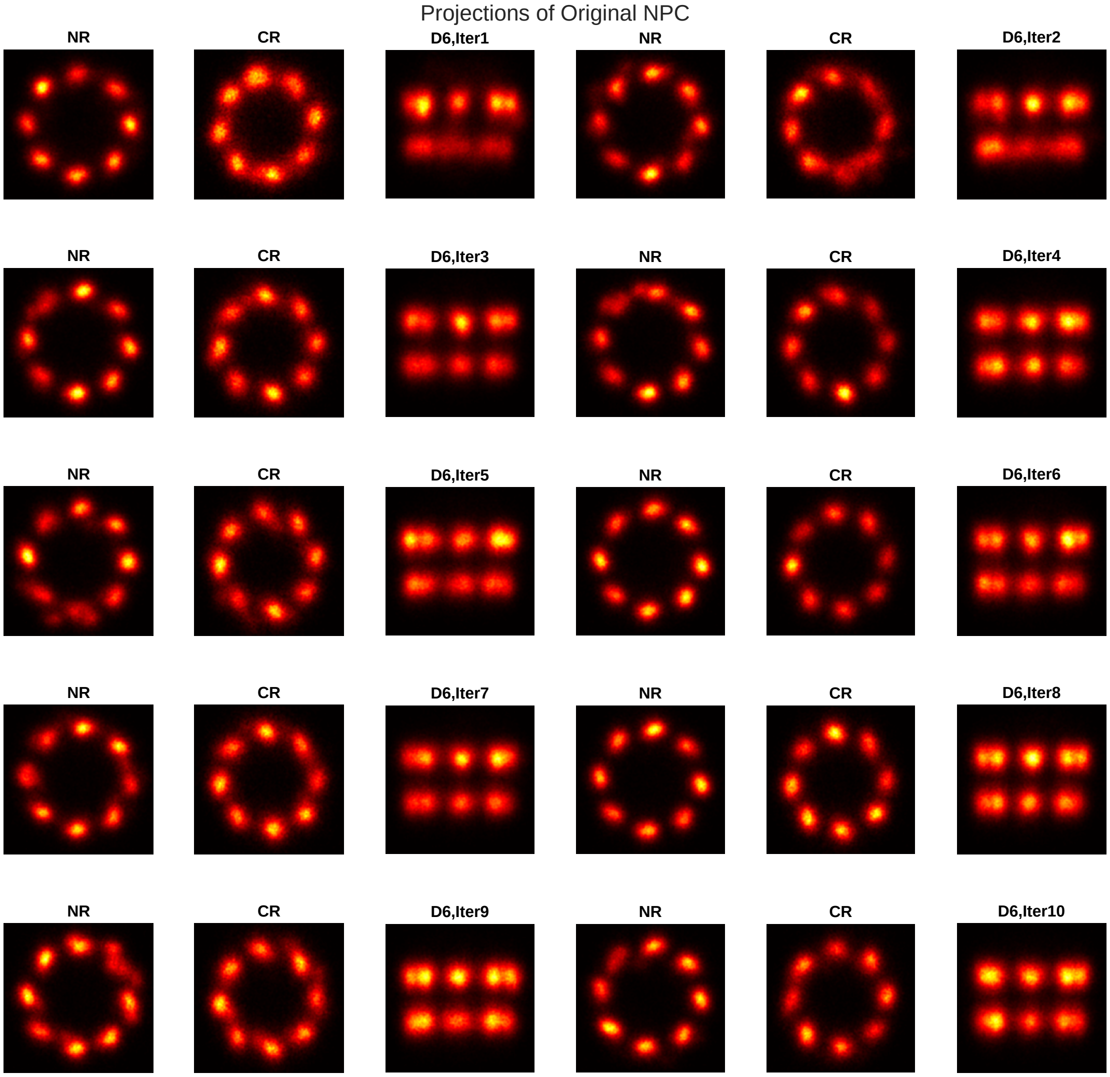

Fig. S6. Data Fusion results for dataset 6 for 10 randomly different GMM initializations without filtering outlier localizations.

B. DATA FUSION RESULTS WITH GMM FILTERING OF OUTLIER LOCALIZATIONS

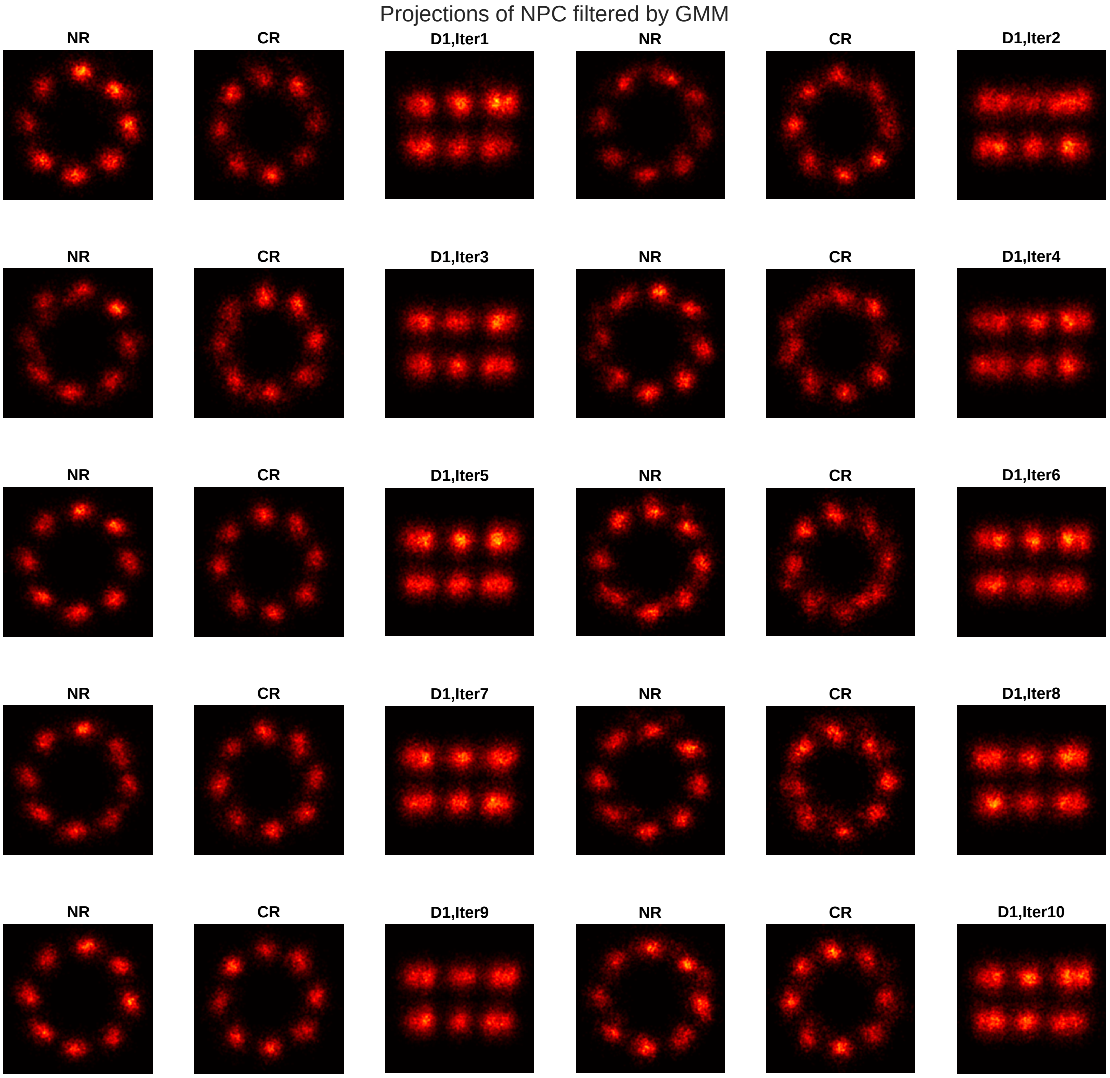

Fig. S7. Data fusion results for dataset 1 for 10 randomly different GMM initializations with GMM filtering of outlier localizations.

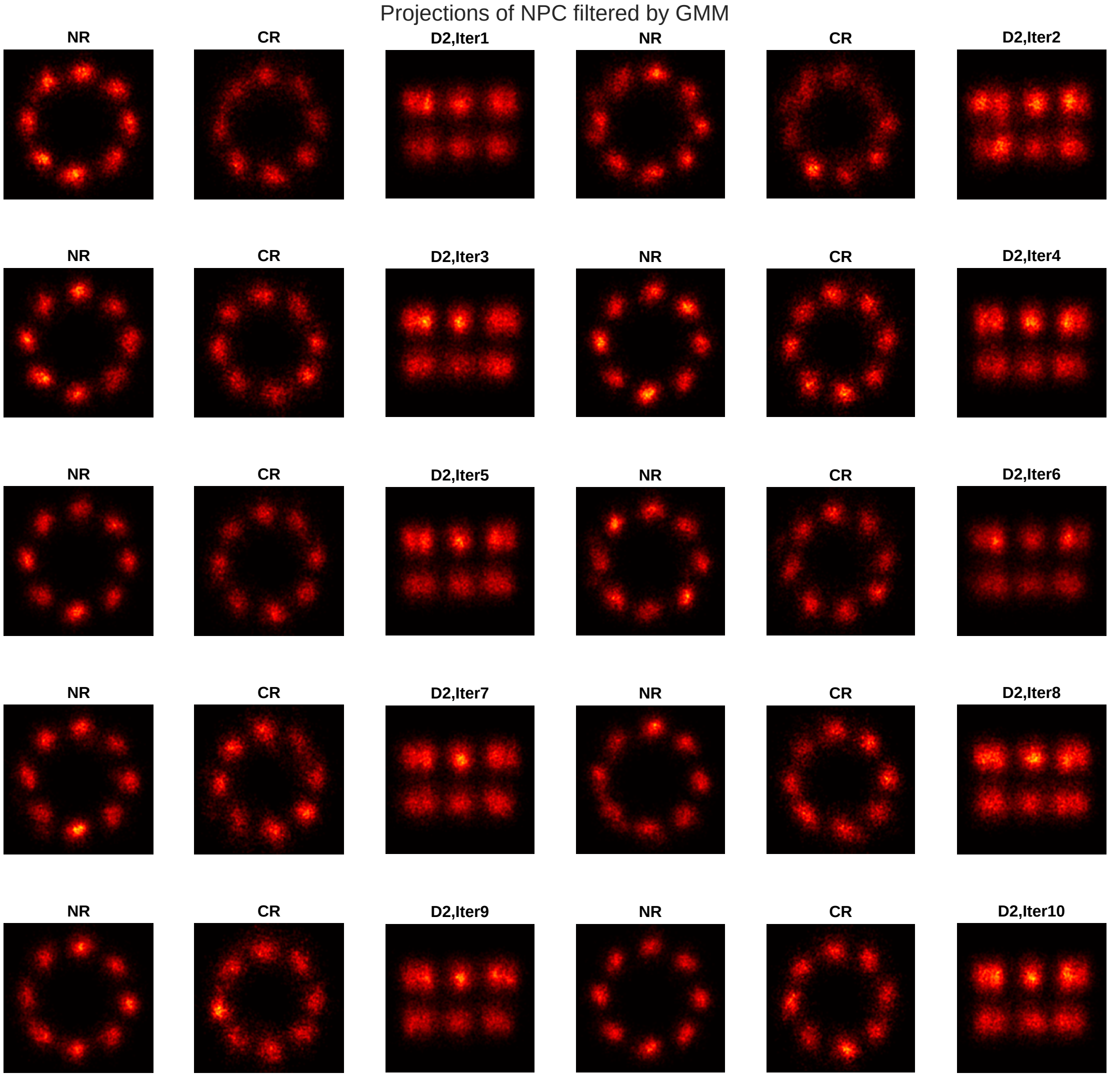

Fig. S8. Data fusion results for dataset 2 for 10 randomly different GMM initializations with GMM filtering of outlier localizations.

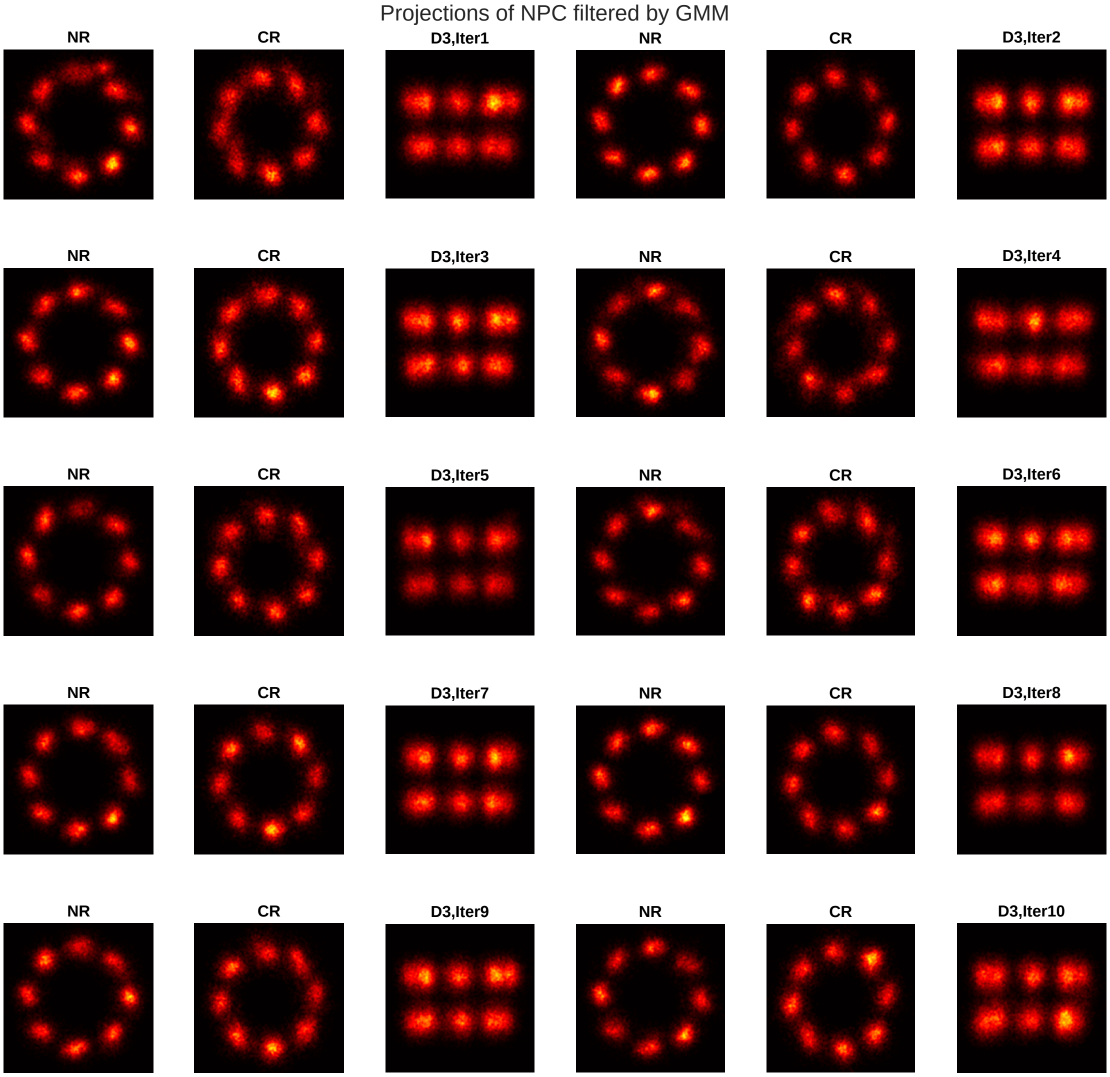

Fig. S9. Data Fusion results for dataset 3 for 10 randomly different GMM initializations with GMM filtering of outlier localizations.

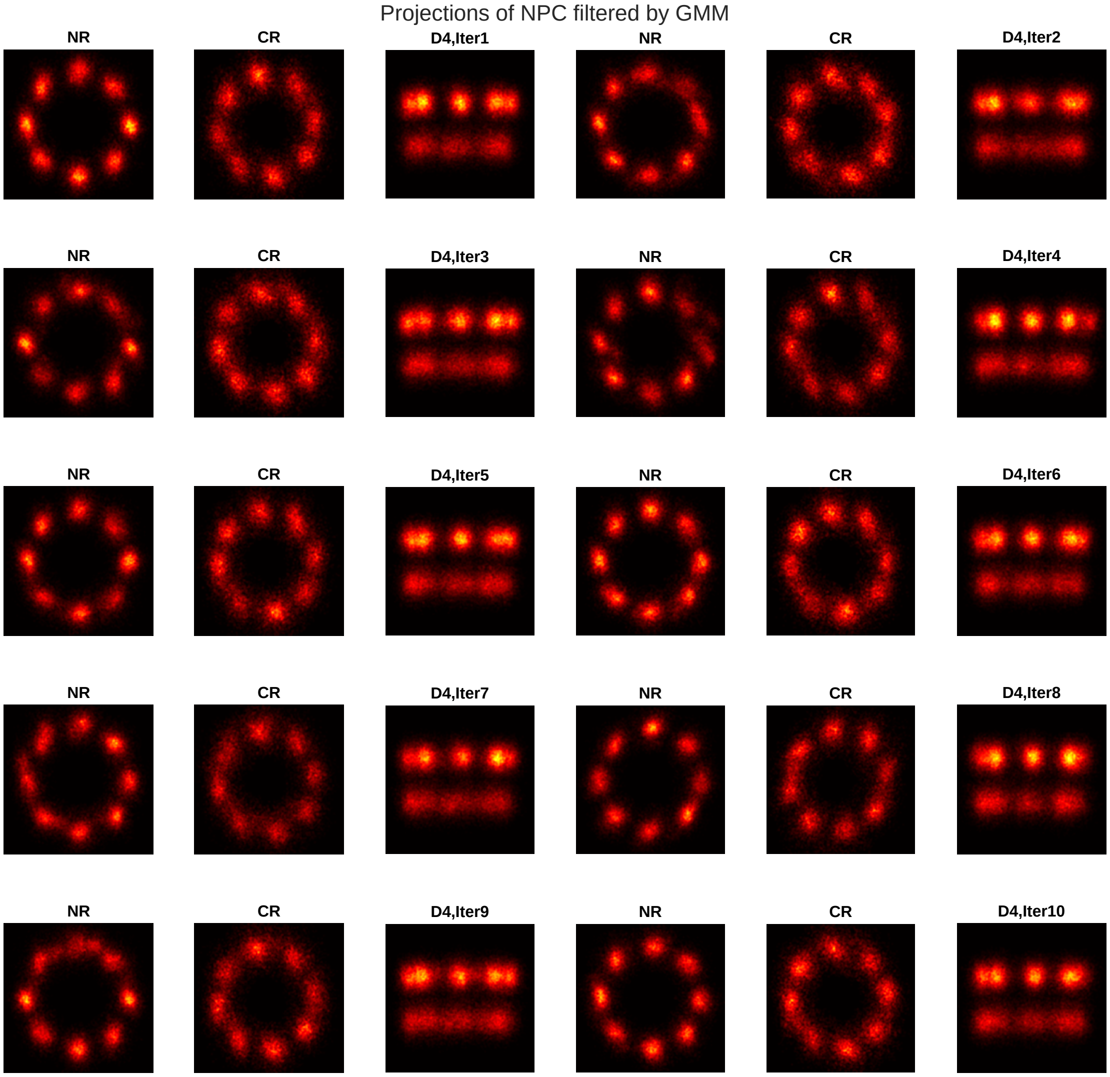

Fig. S10. Data Fusion results for dataset 4 for 10 randomly different GMM initializations with GMM filtering of outlier localizations.

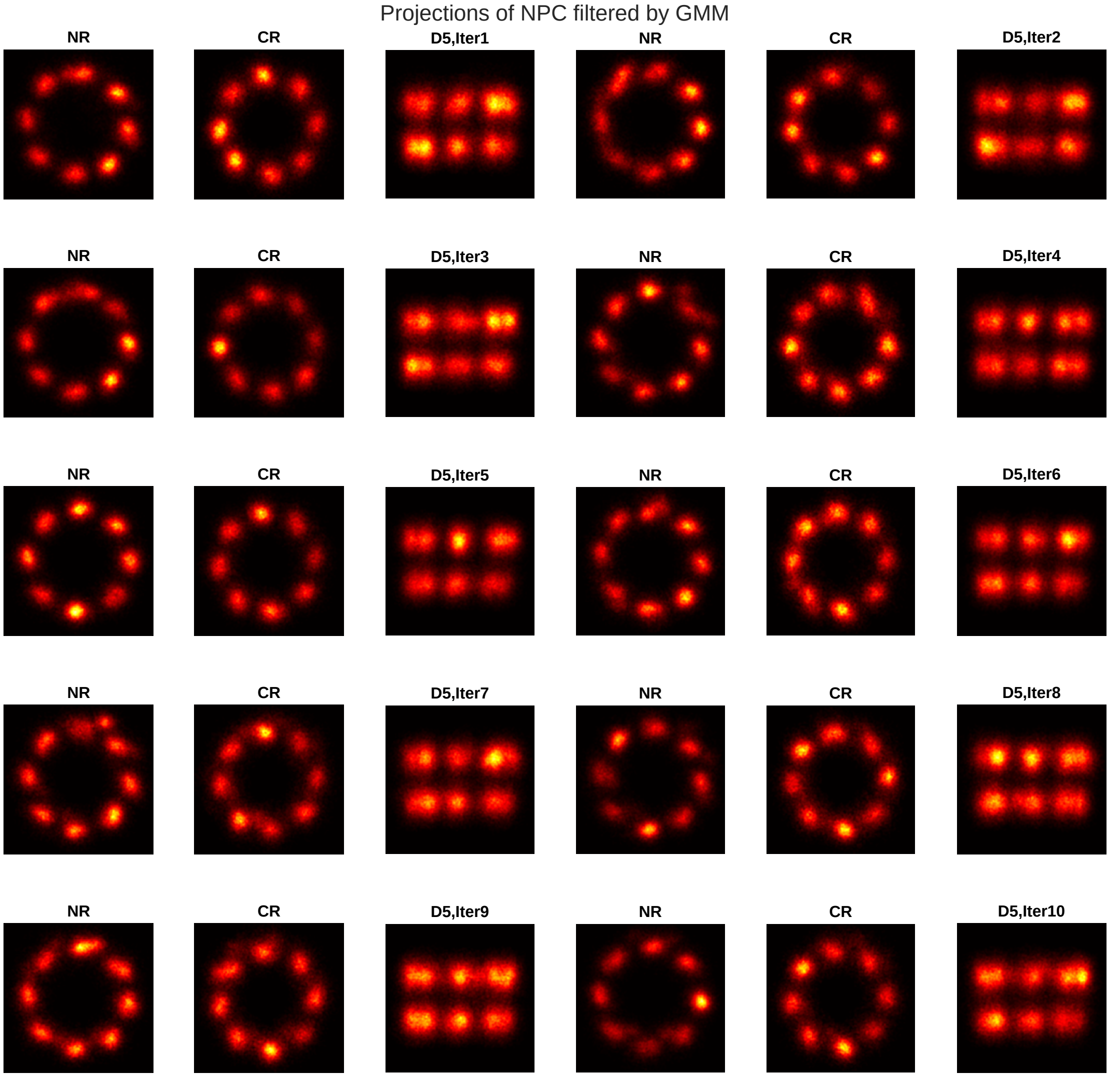

Fig. S11. Data Fusion results for dataset 5 for 10 randomly different GMM initializations with GMM filtering of outlier localizations.

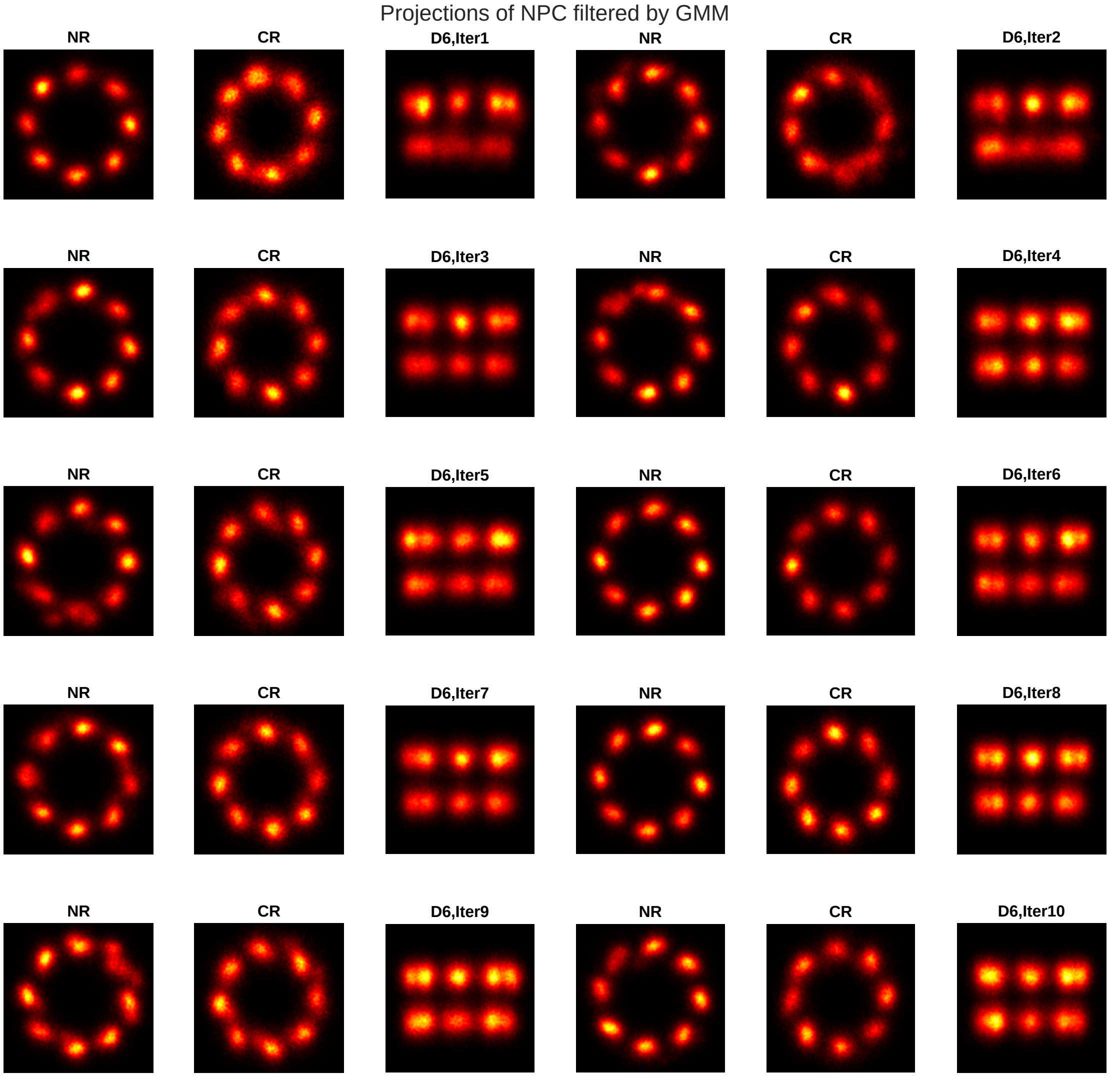

Fig. S12. Data Fusion results for dataset 6 for 10 randomly different GMM initializations with GMM filtering of outlier localizations.

C. DATA FUSION RESULTS WITH GMM AND DENSITY FILTERING OF OUTLIER LOCALIZATIONS

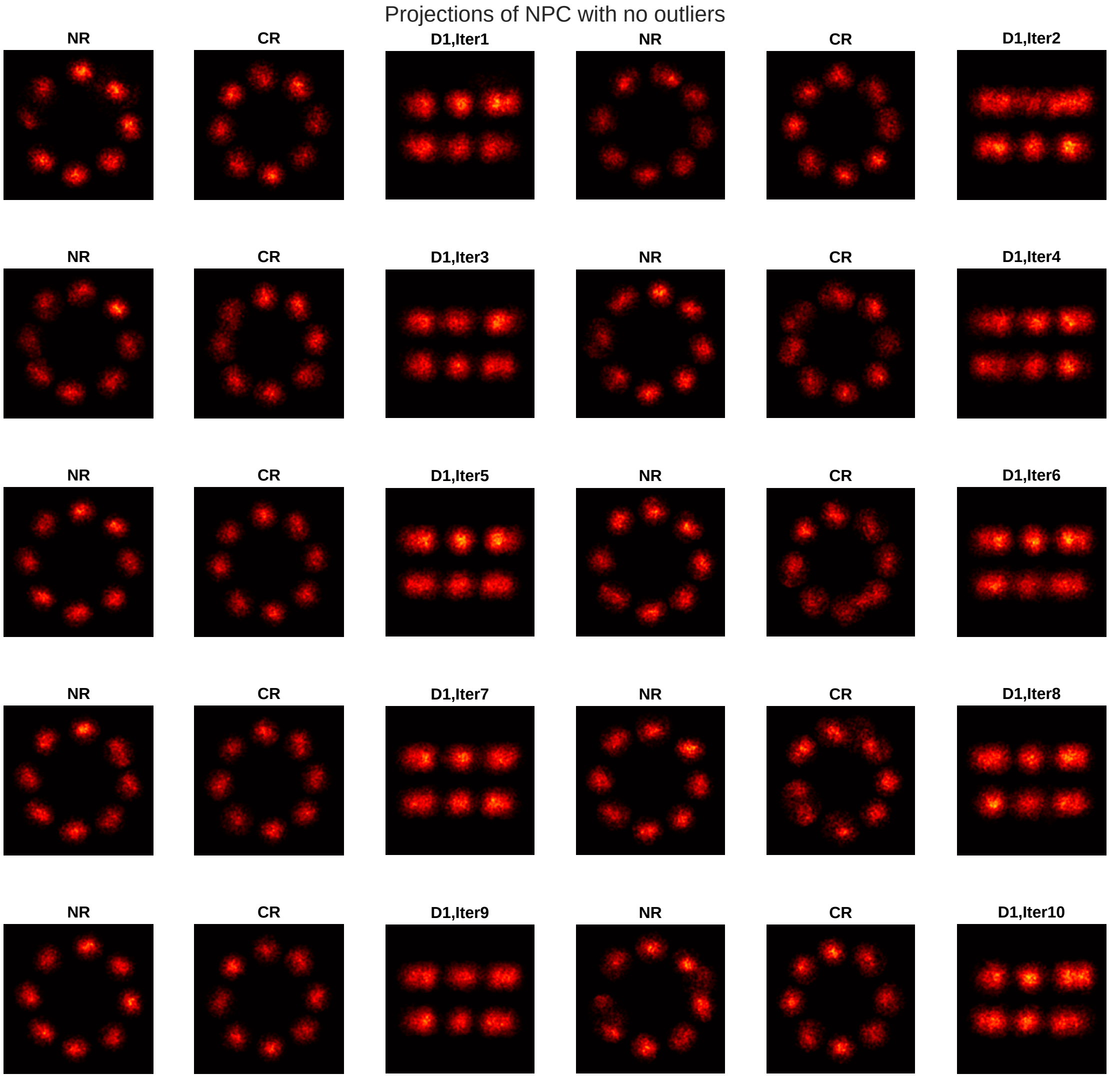

Fig. S13. Data fusion results for dataset 1 for 10 randomly different GMM initializations with GMM and density filtering of outlier localizations.

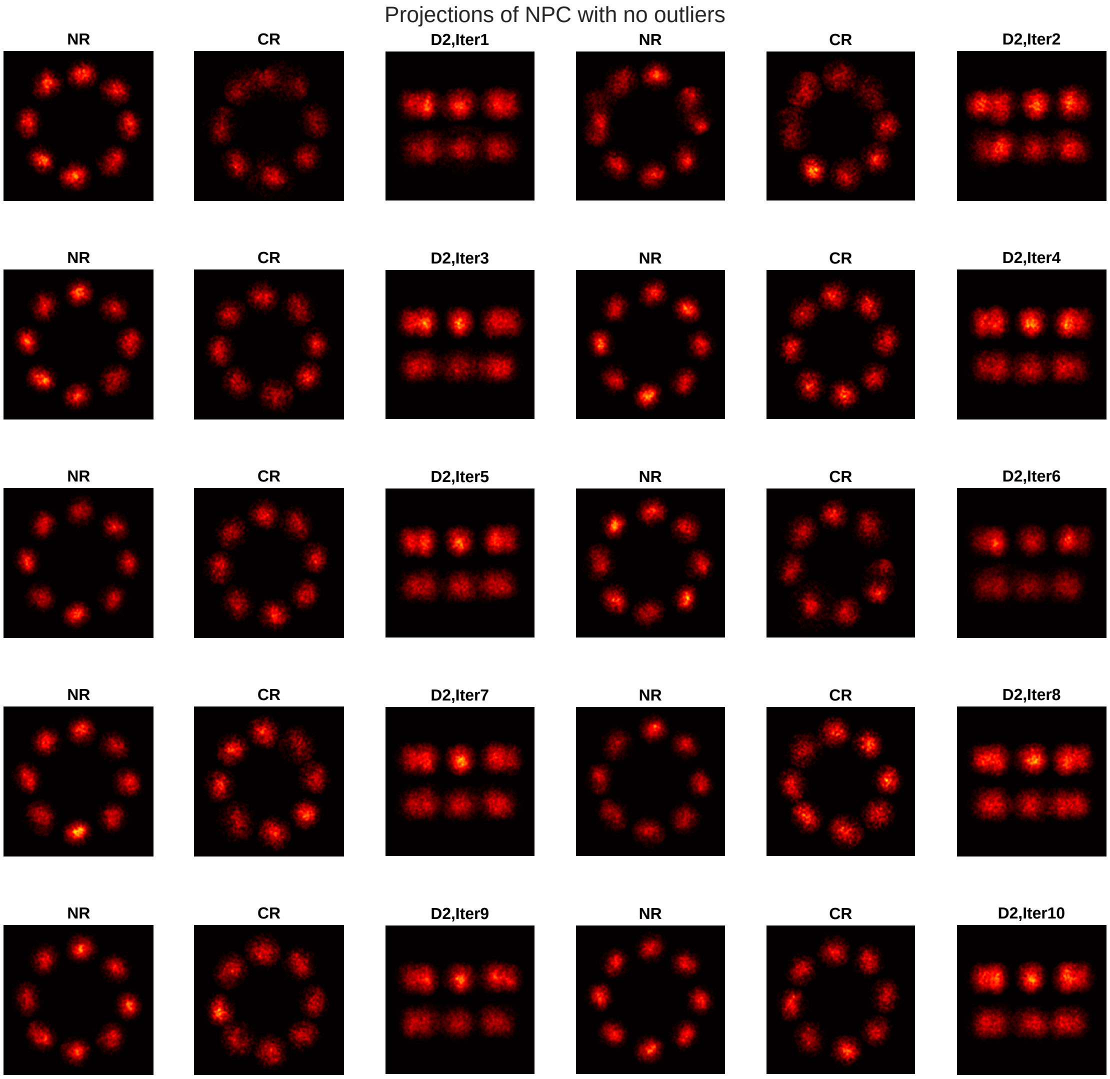

Fig. S14. Data fusion results for dataset 2 for 10 randomly different GMM initializations with GMM and density filtering of outlier localizations.

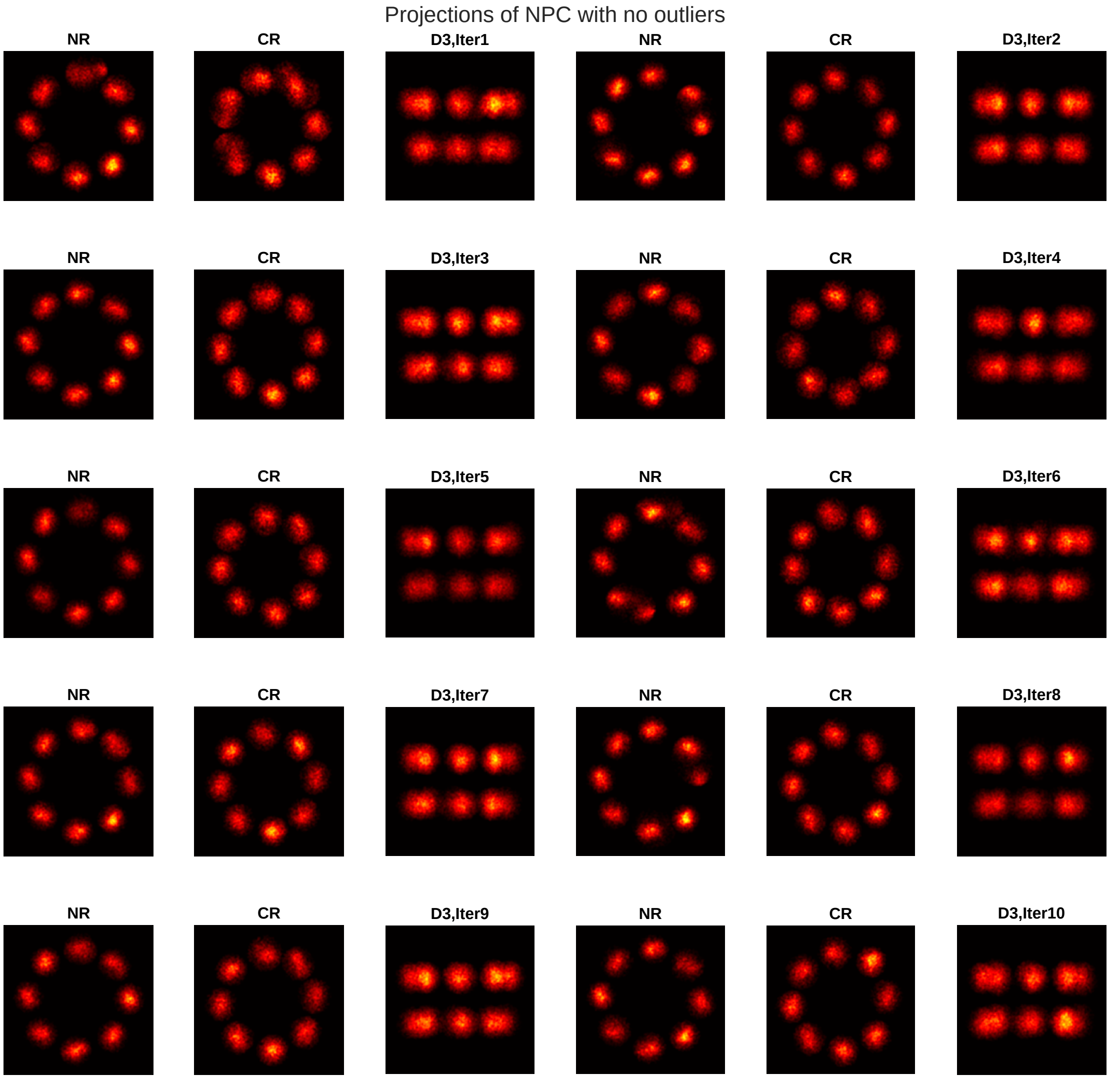

Fig. S15. Data Fusion results for dataset 3 for 10 randomly different GMM initializations with GMM and density filtering of outlier localizations.

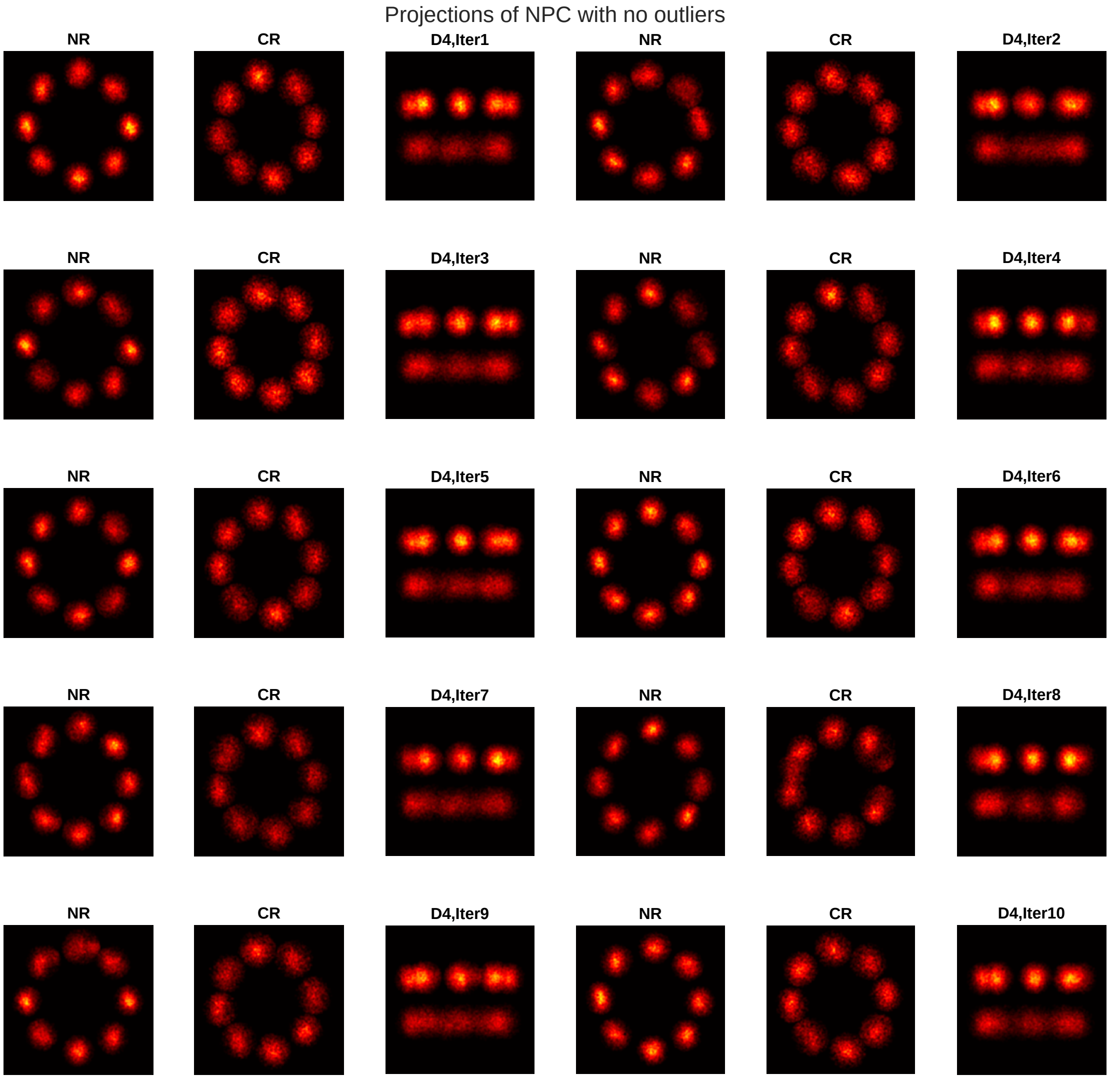

Fig. S16. Data Fusion results for dataset 4 for 10 randomly different GMM initializations with GMM and density filtering of outlier localizations.

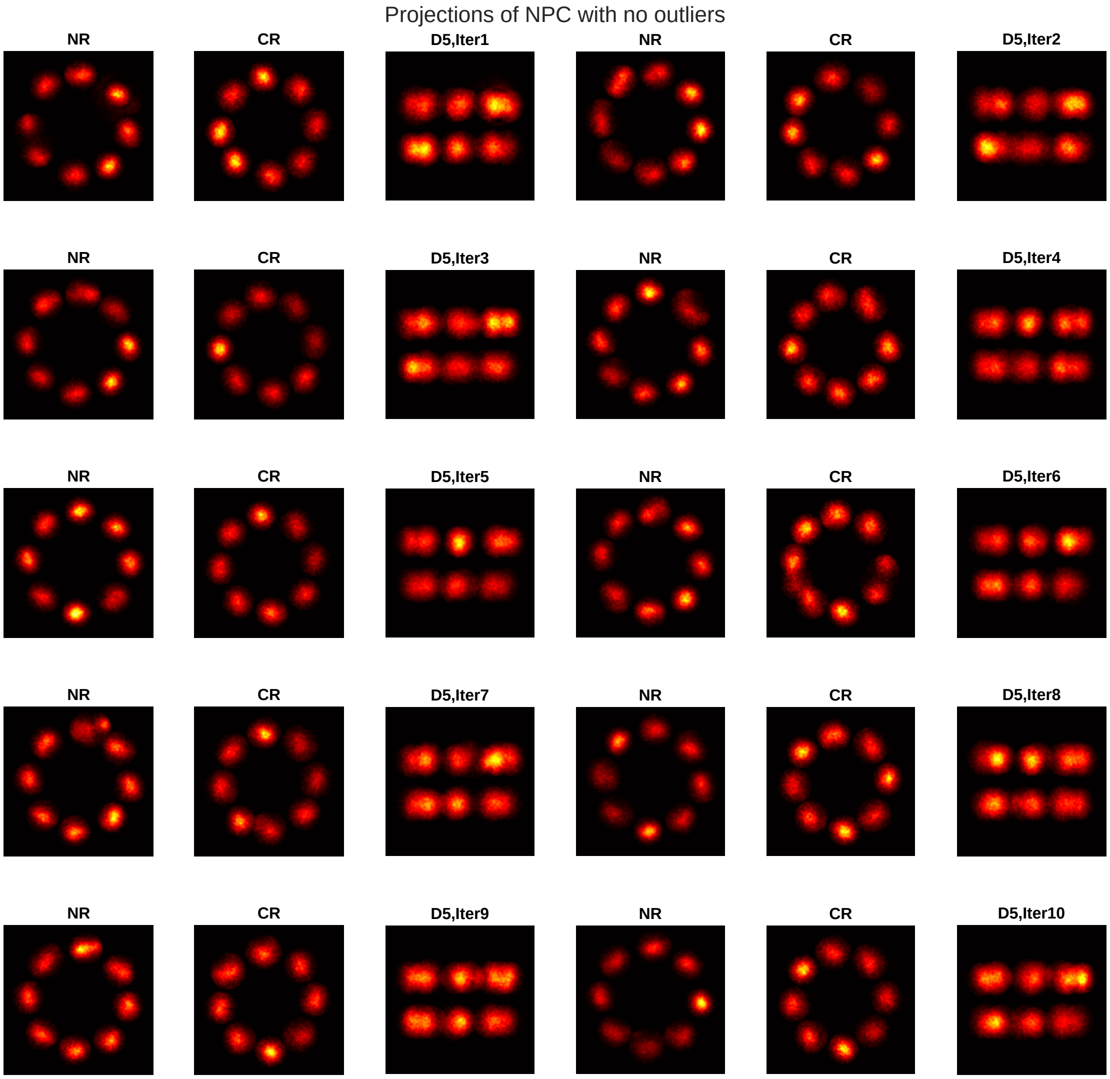

Fig. S17. Data Fusion results for dataset 5 for 10 randomly different GMM initializations with GMM and density filtering of outlier localizations.

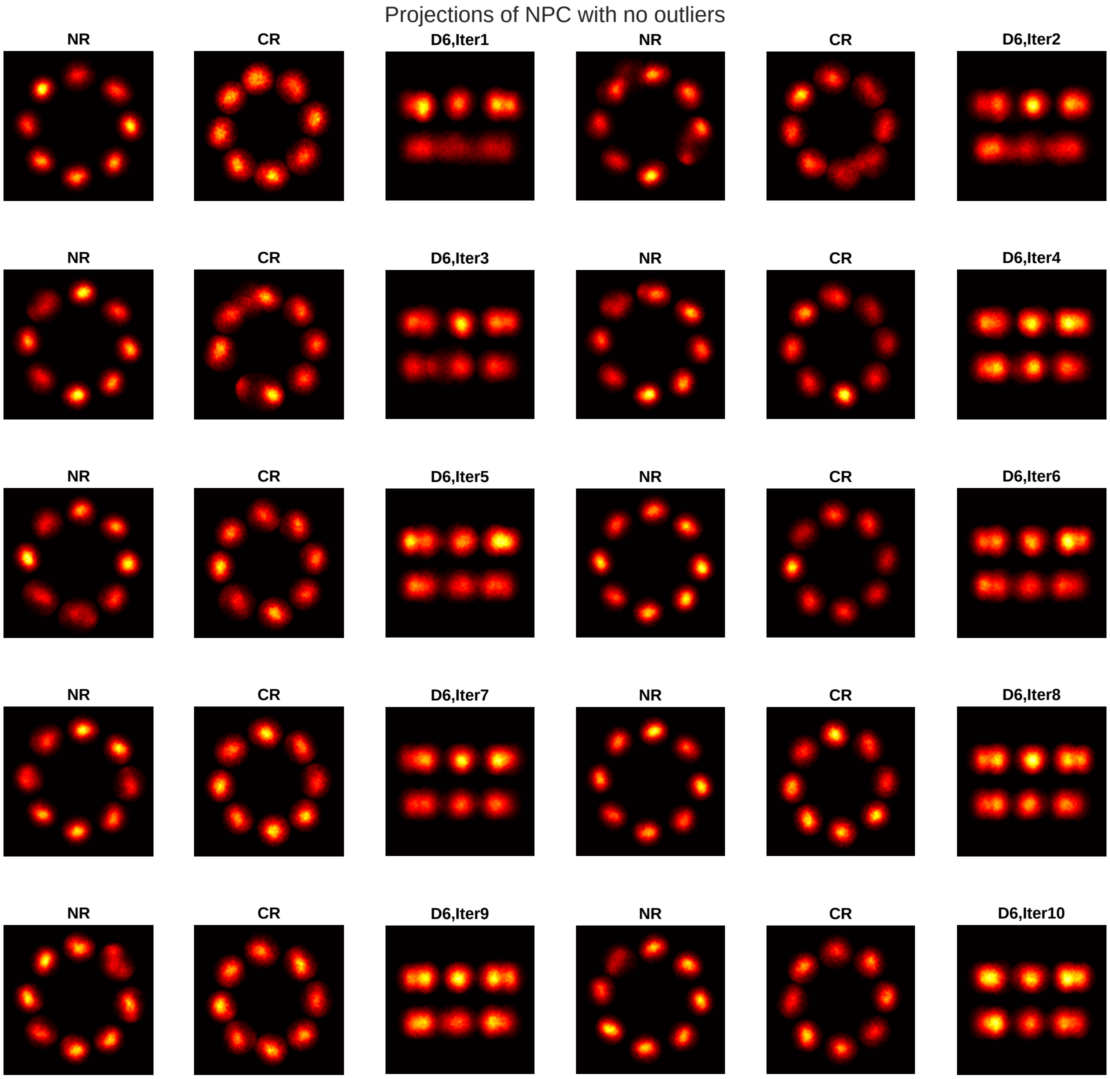

Fig. S18. Data Fusion results for dataset 6 for 10 randomly different GMM initializations with GMM and density filtering of outlier localizations.
